# Phylogenomic analysis and genetic mechanisms of antifungal resistance in clinical isolates of *Candida glabrata* (*Nakaseomyces glabratus*) from across Canada, 2013–2020

**DOI:** 10.1128/spectrum.01803-25

**Published:** 2025-11-26

**Authors:** Domenica G. De Luca, David C. Alexander, Tanis C. Dingle, Philippe J. Dufresne, Jeff Fuller, Greg J. German, David Haldane, Linda M. Hoang, Lei Jiao, Julianne V. Kus, Lisa Li, Kathy Malejczyk, Caroline Sheitoyan-Pesant, Markus Stein, Morag Graham, Gary Van Domselaar, Amrita Bharat

**Affiliations:** 1 National Microbiology Laboratory Branch, Public Health Agency of Canada 41687, Winnipeg, Manitoba, Canada; 2 Department of Medical Microbiology and Infectious Diseases, University of Manitoba 8664, Winnipeg, Manitoba, Canada; 3 Cadham Provincial Laboratory, Diagnostic Services, Shared Health, Winnipeg, Manitoba, Canada; 4 Alberta Precision Laboratories—Public Health Laboratory, Calgary, Canada; 5 Department of Pathology and Laboratory Medicine, University of Calgary 2129, Calgary, Alberta, Canada; 6 Laboratoire de santé publique du Québec, Institut national de santé publique du Québec, Sainte-Anne-de-Bellevue, Québec, Canada; 7 Department of Pathology and Laboratory Medicine, London Health Sciences Centre 10033 , London, Ontario, Canada; 8 Health PEI 420863, Charlottetown, Prince Edward Island, Canada; 9 Department of Laboratory Medicine and Pathobiology, University of Toronto 7938, Toronto, Ontario, Canada; 10 QEII Health Science Centre, Halifax, Nova Scotia, Canada; 11 BC Centre for Disease Control 113269, Vancouver, British Columbia, Canada; 12 Public Health Microbiology Laboratory, St. John’s, Newfoundland and Labrador, Canada; 13 Public Health Ontario 153300, Toronto, Ontario, Canada; 14 Roy Romanow Provincial Laboratory, Regina, Saskatchewan, Canada; 15 Centre hospitalier universitaire Dr-Georges-L.-Dumont, Moncton, Canada; 16 Shared Health, Winnipeg, Manitoba, Canada; Debreceni Egyetem, Debrecen, Hungary

**Keywords:** *Candida glabrata*, *Nakaseomyces glabratus*, whole-genome sequencing, antifungal resistance, phylogenomics, genomic phylogeny, azoles, echinocandins, polyenes, *PDR1*, *FKS1*, *FKS2*

## Abstract

*Candida glabrata* (*Nakaseomyces glabratus*) is an important cause of invasive fungal infections and may exhibit reduced susceptibility toward antifungal drugs. Here, we used whole-genome sequencing to investigate the genomic phylogeny and identify genetic determinants of antifungal resistance in a collection of 142 clinical *C. glabrata* isolates obtained from the 10 provinces of Canada between 2013 and 2020. Our study prioritized resistant isolates (*n* = 62, 43.7%) from invasive infections to better understand antifungal resistance and represents the largest national genomic survey of clinical *C. glabrata* isolates performed to date in Canada. Phylogenomic analysis based on single-nucleotide variants in the core genome revealed 15 genetically related clusters of *C. glabrata*. The largest cluster (cluster I, *n* = 29) was significantly associated with antifungal resistance (*P* value = 0.0112). Antifungal-resistant isolates were present in almost all clusters, suggesting that resistance most likely arises from selective pressure during antifungal therapy, rather than dissemination of resistant clones. Thirty-six unique *PDR1* variants were found in 38/52 (73.1%) fluconazole-resistant *C. glabrata* isolates, with more than half identified in this study as potential novel azole resistance variants. Well-characterized hot-spot variants in *FKS* genes (*n* = 5) were found in 12/13 (92.3%) micafungin-resistant *C. glabrata* isolates. Our genomic analysis highlights the diversity in strain types and sheds light on potential genetic mechanisms of resistance in Canadian isolates of *C. glabrata*.

**IMPORTANCE:**

*Candida glabrata*, also known as *Nakaseomyces glabratus*, is a type of yeast that can cause infections in individuals with weakened immune systems. Invasive infections can be difficult to treat since some *C. glabrata* isolates may not respond well to common antifungals. We studied a large collection of *C. glabrata* isolates collected from across Canada to better understand how *C. glabrata* spreads and why this fungal pathogen sometimes resists treatment. Using whole-genome sequence analysis, we found that drug resistance appears in different strains independently, likely as a result of treatment, rather than spreading from a single clone. We also identified specific mutations that may be linked to resistance to commonly used antifungal drugs, such as fluconazole and micafungin. Our research shows how valuable whole-genome sequencing is for understanding the spread and drug resistance of *C. glabrata*, which can help improve treatment and infection control.

## INTRODUCTION

The yeast *Candida glabrata*, also known as *Nakaseomyces glabratus*, is an opportunistic human fungal pathogen that may cause potentially life-threatening invasive infections. As a commensal member of the human gastrointestinal microbiome, systemic infections of *C. glabrata* most often originate from the intestinal microflora of the host (1, 2), rather than dissemination of clones (3–5)*. C. glabrata* has become a recognized nosocomial pathogen with increasing incidence of invasive disease (3), which is of particular concern since fungal infections by this species are amongst those most often associated with antifungal resistance (6, 7). In many countries worldwide, *C. glabrata* is a leading cause of invasive candidiasis, typically ranking second to *Candida albicans* (7–12). Unlike most *Candida* species, *C. glabrata* displays reduced susceptibility toward azole antifungals, especially fluconazole, and can acquire azole and echinocandin resistance following antifungal therapy (6, 7, 13). Resistance has been reported within all three drug classes in *C. glabrata* and some strains exhibit multidrug resistance (i.e., resistance to two or more drug classes) (7). With only three classes of antifungal drugs (azoles, echinocandins, polyenes) available for the management of invasive infections, resistance to even a single class may severely limit treatment options.

Azole resistance in *C. glabrata* (e.g., fluconazole, itraconazole, posaconazole, and voriconazole) is primarily mediated by amino acid substitutions in *PDR1*, the central transcriptional regulator of efflux pump expression. Activated forms of *PDR1* induce high levels of azole resistance by overexpressing the ATP-binding cassette (ABC) transporters *CDR1*, *PDH1* (*CDR2*), and *SNQ2* (14, 15). Specific regions of *PDR1*, referred to as the inhibitory domain (ID; 312–382 aa), middle homology region (MHR; 539–632 aa), and transcriptional activation domain (TAD; 800–1107 aa), are predictive of azole resistance (16). Alterations in *ERG11*, mitochondrial dysfunction, chromosomal duplications, and ploidy variations have also been reported as minor potential contributors to azole resistance in *C. glabrata* (17–20). Echinocandin resistance (e.g., anidulafungin, caspofungin, and micafungin) in *C. glabrata* is typically mediated by alterations in *FKS* subunits of glucan synthase. Specific positions and amino acid variations in highly conserved hot-spot (HS) regions of *FKS1* and *FKS2* genes are correlated with reduced echinocandin susceptibility (21). The mechanisms of polyene (e.g., amphotericin B) resistance in *C. glabrata* are not well understood; however, genes involved in ergosterol biosynthesis (e.g., *ERG2*, *ERG3*, and *ERG6*) have been implicated (22–24).

Based on a large Canadian multicenter study conducted between 2011 and 2016, *C. glabrata* was the second most common *Candida* species isolated from invasive infections, with resistance rates of 1.0% and 2.5% for fluconazole and micafungin, respectively (8). A study from a large reference laboratory in Ontario, Canada, reported a higher rate of fluconazole resistance (9.0%) during a similar period (25). Although the rates of antifungal resistance in Canada are relatively low, limited information is known beyond the species distribution and phenotypic antifungal susceptibilities of these infections. Several molecular typing methods, such as multi-locus sequence typing (MLST) and microsatellite length polymorphism typing, are widely used to analyze genetic variation in *C. glabrata*; however, whole-genome sequencing provides higher resolution and superior discriminatory power compared to these methods (26).

In this study, we sequenced 142 clinical strains of *C. glabrata* isolated between 2013 and 2020 from the 10 provinces of Canada, representing the largest and most comprehensive genomic study of *C. glabrata* conducted to date in Canada. Invasive infections resistant to at least one antifungal drug from the three major classes (azoles, echinocandins, polyenes) were prioritized to improve insights into antifungal resistance. As such, this collection was not intended to reflect the current rates of antifungal resistance observed in Canada, but rather to investigate antifungal-resistant strains of *C. glabrata* in terms of the genomic phylogeny and associated genetic mechanisms of antifungal resistance.

## MATERIALS AND METHODS

### Isolate collection

The 10 provincial public health laboratories in Canada provided 142 clinical *C. glabrata* isolates collected between 2013 and 2020. We prioritized isolates from invasive infections that were resistant to at least one antifungal drug from the three major drug classes (azoles, echinocandins, and polyenes) to enrich our collection with antifungal-resistant isolates. Twenty-two isolates were from Western Canada (British Columbia or Alberta), 20 from Central West (Saskatchewan or Manitoba), 65 from Central East (Ontario or Québec), and 35 from Eastern Canada (New Brunswick, Newfoundland, Nova Scotia, or Prince Edward Island). Isolates were recovered from sterile and non-sterile body sites, including blood (*n* = 85), urine (*n* = 11), intra-abdominal (*n* = 7), pus (*n* = 7), kidney (*n* = 2), abscess fluid (*n* = 2), bile (*n* = 2), pancreas (*n* = 2), and 24 isolates from various other sites. The line list of isolates used in this study, geographic region of isolation, antifungal minimum inhibitory concentrations (MICs), year, and site of sampling are presented in Table S1.

For the global analysis, we included an additional 139 *C*. *glabrata* strains with antifungal susceptibility data from the National Center for Biotechnology Information (NCBI). Isolates were from Australia (*n* = 50, BioProject PRJNA480138; *n* = 12, PRJNA310957), Belgium (*n* = 6, PRJNA361477), Canada (*n* = 9, PRJNA610214), Denmark (*n* = 8, PRJEB40738), France (*n* = 9, PRJNA361477), Germany (*n* = 1, PRJNA361477; *n* = 2, PRJNA483064), Italy (*n* = 2, PRJNA361477), Switzerland (*n* = 2, PRJNA374542), Taiwan (*n* = 2, PRJNA361477), and the United States (*n* = 20, PRJNA592373; *n* = 12, PRJNA361477; *n* = 3, PRJNA524686; *n* = 1, PRJNA329124). Sequence Read Archive accessions with corresponding BioProject numbers are listed in Table S2.

### Antifungal susceptibility testing

Antifungal susceptibility testing was carried out by broth microdilution either following the Clinical and Laboratory Standards Institute (CLSI) M27-Ed4 guidelines (27) with custom YCML1FCFA panels (Thermo Fisher Oxoid, Oakwood Village, OH) or the manufacturer’s instructions for Sensititre YeastOne Y09 panels (Thermo Oxoid, Oakwood Village, OH). Results for fluconazole, micafungin, and amphotericin B were reported in this study. MICs were evaluated at 24 h and interpreted either using clinical breakpoints established in the CLSI M27M44S Ed3 document (28) (fluconazole and micafungin) or epidemiological cutoff values established in the CLSI M57S Ed4 document (29) (amphotericin B). Resistance breakpoints for fluconazole and micafungin resistance were MICs of ≥64 µg/mL and ≥0.25 µg/mL, respectively. The breakpoint for an intermediate MIC to micafungin was 0.12 µg/mL. The epidemiological cutoff value used for amphotericin B non-wild type was >2 µg/mL. *Candida krusei* ATCC 6258 and *Candida parapsilosis* ATCC 22019 were used as quality control strains.

### Whole-genome sequencing

Genomic DNA (gDNA) was extracted using the MasterPure Complete DNA and RNA Purification Kit (Mandel, Guelph, ON). Libraries were prepared using the Nextera XT DNA Library Preparation kit (Illumina, San Diego, CA) and sequencing was carried out on the NextSeq 500/550 platform (Illumina, San Diego, CA) with the NextSeq 500/550 v2.5 kit (Illumina, San Diego, CA). Isolates with coverage below 60× were re-sequenced.

### Genomic analysis

Raw reads were pre-processed using fastp v.0.23.2 with default settings (30). Phylogenomic analyses based on single-nucleotide variants (SNVs) in the core genome were carried out using the Single Nucleotide Variant Phylogenomic v.1.2.3 pipeline (31). Parameters used were minimum coverage 10, minimum mapping quality 30, SNV abundance ratio 0.75, and SNV density filter >2 SNVs per 20-base window. *C. glabrata* CBS 138 (ATCC 2001) was used as the reference genome (32). Briefly, repeat regions along the reference genome were masked using MUMmer, processed paired-end reads were aligned to the reference genome using SMALT, and SNVs were called by two independent variant callers (FreeBayes and SAMtools/BCFtools). Variant and non-variant calls were merged into a single file and flagged for mismatches between variant callers. Base calls that did not meet the defined thresholds for coverage, mapping quality, and SNV abundance were removed. High-density regions were identified to mask potential recombinant regions. PhyML was used to construct a maximum-likelihood phylogeny based on the core genome SNV alignment using the GTR + γ substitution model. The SNV alignment was also used to generate a pairwise SNV distance matrix. Maximum-likelihood dendrograms were visualized and annotated using the Interactive Tree of Life v.7.2 (33).

For the variant analysis, the processed reads were aligned using BWA-mem v.0.7.17.1 (34) and sorted using SAMtools v.1.18 (35). Variants were called using freebayes v.1.3.7 (minimum mapping quality: 30, minimum coverage: 10, minimum alternate allele fraction: 0.75, minimum base quality: 20) (36). snpEff v.5.2 (37) was used to annotate variants and snpSIFT v.5.2 (38) was applied to extract the resistance genes of interest.

Copy number variants (CNVs) were identified using a read-depth approach. GC bias was corrected using deepTools computeGCbias v.3.3.2.0.0 and correctGCbias v.3.3.2.0.0 (39). The reference genome was divided into 1,000 base pair windows using BEDTools makewindows v.2.31.1 (40). Read coverage across the genome was calculated from aligned and sorted BAM files using BEDTools genomecov v.2.31.1 (40). The average read depth per window was then computed using bedtools map v.2.31.1 (40). Chromosome-end bias was corrected based on the average read depth versus distance to the nearest chromosome end using a custom R script for locally weighted scatterplot smoothing regression (41). Coverage values were normalized by dividing by the genome-wide median coverage. Genes with normalized coverage values ≥2 or 0 were considered to have increased or decreased copy numbers, respectively. CNVs were subsequently identified, annotated, and visualized using a custom R script (41).

### Statistical analysis

The Fisher’s exact test was used to determine if there were statistically significant associations between clusters and categorical variables, such as region of isolation, body site of isolation, and antifungal resistance. *P* values of <0.05 were considered statistically significant.

## RESULTS

### Antifungal susceptibility testing

MICs for fluconazole, micafungin, and amphotericin B were determined for all isolates in the collection by broth microdilution (Table 1). Fifty-two isolates (36.6%) were resistant to fluconazole (MIC ≥ 64 µg/mL), 13 (9.2%) were resistant to micafungin (MIC ≥ 0.25 µg/mL), and a single isolate (0.7%) was non-wild type to amphotericin B (MIC > 2 µg/mL). In total, 62 (43.7%) *C*. *glabrata* isolates were resistant to at least one antifungal drug (fluconazole, micafungin, or amphotericin B). Four isolates were multidrug resistant to two drug classes (fluconazole + micafungin). Four isolates had intermediate MICs to micafungin (MIC = 0.12 µg/mL).

**TABLE 1:** Distribution of MICs of fluconazole, micafungin, and amphotericin B against Canadian isolates of *C. glabrata* (*n* = 142)^*a*^

Antifungal drug	MIC (µg/mL)																		
	≤0.008	0.016	0.03	0.06	0.12	0.25	0.5	1	2	4	8	16	32	64	128	>128	% S/SDD^*b*^/WT^*c*^	% I	% R/NWT^*c*^
FLZ	NA	NA	NA	NA	NA	NA	1	2	4	24	42	13	4	**19**	**23**	**10^*d*^**	63.4	NA	36.6
MFG	49	56	13	7^*e*^	4	**2**	**1**	**3**	**4**	**2**	**1**	NA	NA	NA	NA	NA	88.0	2.8	9.2
AMB	NA	NA	NA	NA	4	19^*f*^	59	46	13	**1**	NA	NA	NA	NA	NA	NA	99.3	NA	0.7

^*a*^ MIC, minimum inhibitory concentration; FLZ, fluconazole; MFG, micafungin; AMB, amphotericin B; S, susceptible; SDD, susceptible dose-dependent; WT, wild type; I, intermediate; R, resistant; NWT, non-wild type; NA, not applicable. Boldface indicates resistance and/or non-wild type (FLU, ≥64 µg/mL; MFG, 0.25 µg/mL; AMB, >2 µg/mL).

^*b*^ SDD category only for fluconazole.

^*c*^ WT and NWT categories only for amphotericin B.

^*d*^ Includes 2 isolates at 256 and 1 isolate at > 256.

^*e*^ Includes 2 isolates at < 0.06.

^*f*^ Includes 1 isolate at < 0.25.

**TABLE 2:** Association between clusters and antifungal resistance for Canadian isolates of *C. glabrata^a^*

Cluster (*n*)	S/SDD^*b*^/WT^*c*^ isolates (%)	R/NWT^*c*^ isolates (%)	*P* value
I (29)	10 (34.5)	19 (65.5)	**0.0112**
II (3)	2 (66.7)	1 (33.3)	1.0000
III (22)	15 (68.2)	7 (31.8)	0.2509
IV (4)	3 (75.0)	1 (25.0)	0.6319
V (3)	1 (33.3)	2 (66.7)	0.5806
VI (4)	2 (50.0)	2 (50.0)	1.0000
VII (6)	3 (50.0)	3 (50.0)	1.0000
VIII (12)	7 (58.3)	5 (41.7)	1.0000
IX (6)	3 (50.0)	3 (50.0)	1.0000
X (15)	10 (66.7)	5 (33.3)	0.4263
XI (2)	2 (100.0)	0 (0.0)	0.5045
XII (2)	2 (100.0)	0 (0.0)	0.5045
XIII (3)	2 (66.7)	1 (33.3)	1.0000
XIV (4)	1 (25.0)	3 (75.0)	0.3182
XV (15)	9 (60.0)	6 (40.0)	0.7916

^*a*^ S, susceptible; WT, wild type; R, resistant; NWT, non-wild type. Boldface indicates statistically significant association between cluster and antifungal resistance (*P* value < 0.05).

^*b*^ Susceptible dose-dependent category only for fluconazole.

^*c*^ Wild-type and non-wild-type categories only for amphotericin B only.

**TABLE 3:** *PDR1* variants identified in fluconazole-resistant *C. glabrata* (*n* = 38) isolates^*a*^

Cluster	Isolate	Region	FLZ MIC (µg/mL)	Confirmed *PDR1* variant^*b*^	Suspected *PDR1* variant^*c*^	Potential novel *PDR1* variant^*d*^
I	MYC-19-0087	Central East	128		G583S (16, 42)	
	MYC-19-0093	Central East	128			K1087E
	MYC-19-0181	West	128		G583S (16, 42)	
	MYC-19-0275	Central West	128			S343P
	MYC-19-0317	Central East	64		G583S (16, 42)	
	MYC-19-0318	Central East	64		E340K (43, 44)	
	MYC-19-0324	Central East	64		D1089Y (16)	
	MYC-19-0326	Central East	64	G1099D (45)		
	MYC-19-0334	Central East	128		G583S (16, 42)	
	MYC-19-0335	Central East	>128			A693E
	MYC-19-0338	Central East	128			L291F
	MYC-19-0341	Central East	>128			L931F
	MYC-19-0343	Central East	128			C350R
II	MYC-19-0332	Central East	128		S942F (46, 47)	
III	MYC-19-0015	East	64			N764I
	MYC-19-0083	Central East	128			N764I
	MYC-19-0161	West	>128			E1083G
	MYC-19-0339	Central East	128	G346D (45, 48)		
IV	MYC-19-0174	West	64		S316I (16, 42–44, 46)	
V	MYC-19-0340	Central East	64		T360I (16)	
VI	MYC-19-0274	Central West	64			P235L
VII	MYC-19-0319	Central East	128	D876Y (16)		
	MYC-19-0320	Central East	128		D876N (49)	
	MYC-19-0346	Central East	64		R761S (46)	
VIII	MYC-19-0092	Central East	≥256			I616F
	MYC-19-0166	Central West	128			W256R
	MYC-19-0330	Central East	64			Y124S
IX	MYC-19-0088	Central East	≥256		R592S (16, 42)	
	MYC-19-0238	East	64			I207T
	MYC-19-0273	Central West	128		D876N (49)	G611V
X	MYC-19-0297	East	128		D876N (49)	
	MYC-19-0336	Central East	128		K274E (50)	F613del
	MYC-19-0342	Central East	64		T1080A (42)	
XV	MYC-19-0025	East	64			R761G
	MYC-19-0089	Central East	256			V849L
–^*e*^	MYC-19-0082	Central East	128		R772K (16, 43, 44)	I971V
–^*e*^	MYC-19-0325	Central East	64			L1081F
–^*e*^	MYC-19-0345	Central East	64		G1079R (16, 43, 51)	G189V

^*a*^ FLZ, fluconazole; MIC, minimum inhibitory concentration.

^*b*^ Confirmed *PDR1* variants are defined as those that were previously experimentally verified to confer azole resistance in *C. glabrata*.

^*c*^ Suspected *PDR1* variants are defined as those found in at least one other azole-resistant *C. glabrata* isolate from a previous study but not experimentally verified to confer azole resistance.

^*d*^ Potential novel *PDR1* variants are defined as those that were identified only in azole-resistant *C. glabrata* isolates in this study for the first time.

^*e*^ “–” indicates that the isolate did not belong to a cluster.

### Phylogenomic analysis

**Fig 1:**
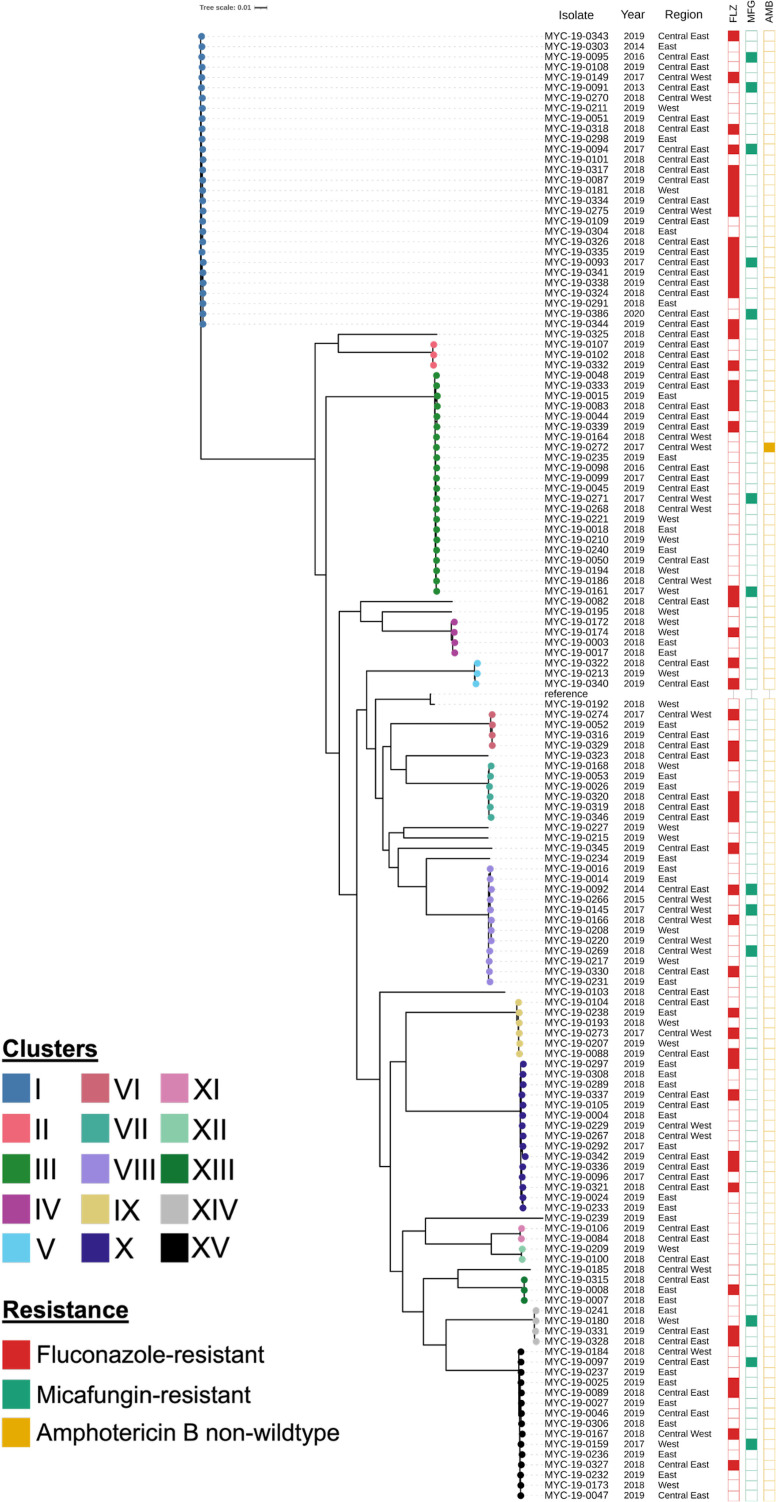
Maximum-likelihood phylogeny of *C. glabrata* isolates from Canada (*n* = 142) based on SNVs in the core genome. This phylogeny represents 75.5% of the core genome of reference strain CBS 138 (ATCC 2001). The 15 clusters (defined as two or more isolates <1,000 SNVs apart) are represented in various colors. Metadata includes the year of isolation, region of isolation, and resistance/non-wild type to fluconazole (red), micafungin (green), and/or amphotericin B (yellow). Antifungal-resistant/non-wild-type isolates are indicated by colored boxes.

Phylogenomic analysis was carried out based on SNVs in the core genome (Fig. 1). Overall, the phylogeny represented 75.5% of the *C. glabrata* reference strain CBS 138 (ATCC 2001) genome. Most isolates (130/142, 91.5%) grouped into one of 15 genetically related clusters. A cluster was defined as two or more isolates that were less than 1,000 SNVs apart. Isolates within clusters differed by <10 to hundreds of SNVs (Table S3). Isolates between clusters differed by >13,500 SNVs, except for clusters XI and XII, which each differed by approximately 7,000 SNVs from the next most related isolate (Table S3). The five largest clusters (cluster I, *n* = 29; cluster III, *n* = 22; cluster VIII, *n* = 12; cluster X, *n* = 15; and cluster XV, *n* = 15) consisted of more than half (65.5%) of the isolates in our collection.

There were several clusters that contained closely genetically related isolates (defined in this study as ≤20 SNVs). In cluster I (*n* = 29, 2-526 SNVs), there were three fluconazole-resistant isolates (MYC-19-0324, MYC-19-0341, and MYC-19-0344) and one micafungin-resistant isolate (MYC-19-0386) from the same province in the Central East region of Canada that differed by 2-16 SNVs (Fig. 1). Two of these isolates (MYC-19-0344 and MYC-19-0386) were collected from the same patient with differing susceptibility profiles and were separated by only two SNVs. The remaining isolates (MYC-19-0324 and MYC-19-0341) were recovered from separate patients at different healthcare facilities. A susceptible isolate (MYC-19-0291) from the Eastern region of Canada was also closely related to this group (8-14 SNVs). Within cluster VIII (*n* = 12, 2-681 SNVs), there were two susceptible isolates (MYC-19-0208 and MYC-19-0220) from the West and Central West regions of Canada separated by 3 SNVs (Fig. 1). There were several other instances of closely genetically related isolate pairs from the same patient (cluster III, MYC-19-0044 and MYC-19-0339, 3 SNVs; cluster VIII: MYC-19-0014 and MYC-19-0016, 2 SNVs; cluster X: MYC-19-0024 and MYC-19-0233, 13 SNVs; and cluster XIII, MYC-19-0007 and MYC-19-0008, 2 SNVs) observed in our study (Fig. 1).

Clusters did not group based on region of isolation within Canada (Fig. 1). Two potential minor exceptions were determined in clusters II and XI (Fig. 1). Isolates in cluster II (*n* = 3, 179-211 SNVs) were from the Central East region of Canada but from different provinces, while the two strains in cluster XI (*n* = 2, 297 SNVs) were from the same province in the Central East region of Canada; however, these associations were not significant (cluster III, *P* value = 0.0935; cluster XI, *P* value = 0.2078).

Some clusters appeared to be enriched for bloodstream or sterile site isolates. All strains in clusters IX (*n* = 6, 181-554 SNVs), XI (*n* = 2, 297 SNVs), and XII (*n* = 2, 200 SNVs) were isolated from blood (i.e., sterile site) (Table S1). Isolates in clusters II (*n* = 3, 179-211 SNVs) and XIII (*n* = 3, 2-37 SNVs) were collected from various sterile body sites (cluster II, bile = 1, blood = 2; cluster XIII, abdomen fluid = 1, blood = 2) (Table S1). These associations, however, were not statistically significant.

The majority of antifungal-resistant *C. glabrata* isolates (58 of 62, 93.5%) were present in 13 of 15 (86.7%) clusters (Fig. 1). Clusters XI (*n* = 2) and XII (*n* = 2) were the only clusters that did not contain at least one resistant isolate. Cluster I had the greatest number of antifungal-resistant isolates with 19/29 (65.5%) resistant to at least one antifungal drug (fluconazole, *n* = 14; micafungin, *n* = 3; and fluconazole + micafungin, *n* = 2) (Table 2). The associations of cluster I with antifungal resistance (*P* value = 0.0112) and fluconazole resistance (*P* value = 0.0298) were statistically significant, but not micafungin resistance (*P* value = 0.1403). Cluster III had the second largest number of antifungal-resistant isolates with 7/22 (31.8%) resistant to at least one antifungal drug (fluconazole, *n* = 4; micafungin, *n* = 1; amphotericin B, *n* = 1; and fluconazole + micafungin, *n* = 1); however, this cluster was not significantly associated with antifungal resistance (*P* value = 0.2509) or fluconazole resistance (*P* value = 0.1578) or amphotericin B resistance (*P* value = 0.1549) (Fig. 1). Fluconazole-resistant isolates (*n* = 52) were found in all clusters except for clusters XI and XII (Fig. 1). All micafungin-resistant isolates (*n* = 13) fell into one of five clusters (cluster I, *n* = 5; cluster III, *n* = 2; cluster VIII, *n* = 3; cluster XIV, *n* = 1, and cluster XV, *n* = 2) (Fig. 1). The sole amphotericin B-resistant isolate was found in cluster III (Fig. 1). There were four fluconazole-resistant *C. glabrata* isolates that did not cluster, highlighting that resistance was widely distributed within the phylogeny (Fig. 1).

### Global phylogenomic analysis

**Fig 2:**
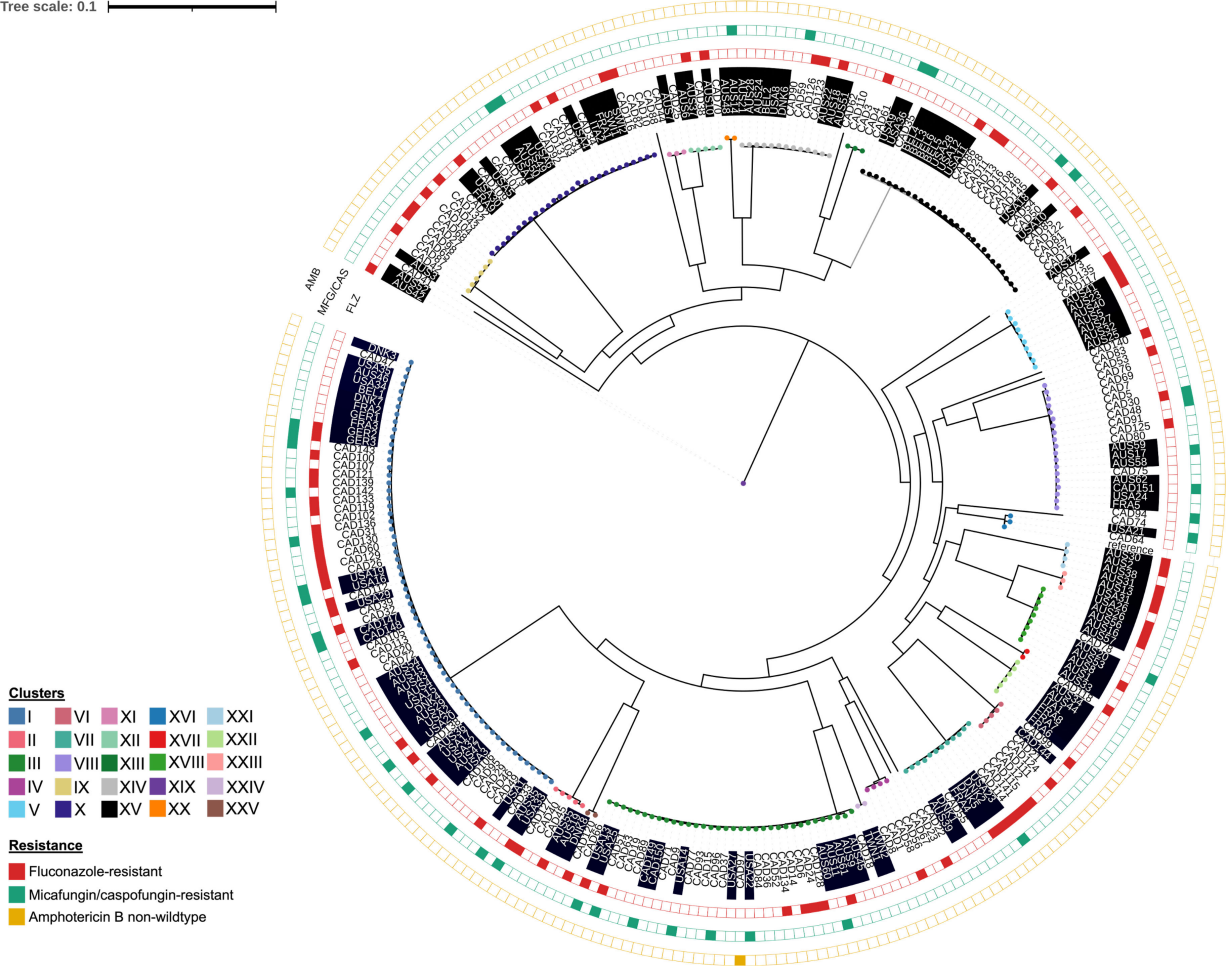
Maximum-likelihood phylogeny of global *C. glabrata* isolates (*n* = 281) based on SNVs in the core genome. The global analysis includes isolates (highlighted in black) from 10 countries (Australia [AUS], *n* = 62; Belgium [BEL], *n* = 6; Canada [CAD], *n* = 151; Denmark [DNK], *n* = 8; France [FRA], *n* = 9; Germany [GER], *n* = 3; Italy [ITA], *n* = 2; Switzerland [SWI], *n* = 2; Taiwan [TWN], *n* = 2; and the United States [USA], *n* = 36) with susceptibility data for fluconazole, micafungin/caspofungin, and/or amphotericin B. Isolates resistant/non-wild type to fluconazole (red), micafungin/caspofungin (green), and/or amphotericin B (yellow) are represented in colored boxes.

We added 139 *C. glabrata* isolates from NCBI to our collection to analyze the Canadian isolates within a broader global context (Table S2) (25, 52–55). In total, there were 281 *C. glabrata* isolates from 10 countries (Australia, *n* = 62; Belgium, *n* = 6; Canada, *n* = 151; Denmark, *n* = 8; France, *n* = 9; Germany, *n* = 3; Italy, *n* = 2; Switzerland, *n* = 2; Taiwan, *n* = 2; and the United States, *n* = 36) included in the global phylogenomic analysis based on SNVs in the core genome. Since only isolates with reported MICs for fluconazole, micafungin, caspofungin, and/or amphotericin B were included, our global phylogeny is biased toward Canadian isolates, with more than half (53.7%) from Canada. Cluster labels from the Canadian phylogeny were applied to the global phylogeny using the same cluster definition of two or more isolates <1,000 SNVs. Of the 281 *C. glabrata* isolates, 271 (96.4%) grouped into one of 25 clusters that ranged in size from two to 63 isolates (Fig. 2). The isolates (PRJNA361477, Table S2) used to support the existence of seven major global clades *of C. glabrata* were included in our global analysis and were distributed across only eight of the clusters we observed using our <1,000 SNV cutoff (52). While it is challenging to compare lineages defined across studies due to the lack of standardized criteria, our global phylogenomic analysis highlights that the global diversity of *C. glabrata* exceeds previous reports.

Eight clusters of the global *C. glabrata* phylogeny grouped according to country (Australia: clusters XIX, XX, and XXI; Canada: clusters IV, VI, IX, and XIII; and Taiwan: cluster XXIV) (Fig. 2). Several of these clusters were significantly associated with geographic region of isolation (cluster IX, *P* value = 0.0322; cluster XXIV, *P* value = 0.0022; cluster XIX, *P* value = 0.0481; cluster XX, *P* value = 0.0481; and cluster XXI, *P* value = 0.0022).

Eighty-three isolates were resistant to azoles, 38 were resistant to echinocandins, and one was resistant to amphotericin B. Isolates resistant to at least one antifungal drug were found in 22 of 25 clusters (Fig. 2). No resistant isolates were present in clusters XI, XXII, and XXIV (Fig. 2). Similar to the Canadian phylogeny, clusters I and III were enriched with antifungal-resistant isolates (Fig. 2). In cluster I, 32/63 (50.8%) of isolates were resistant to at least one antifungal drug, while 13/34 (38.2%) were resistant in cluster III. The association of cluster I with antifungal resistance was not maintained at the global level (*P* value > 0.05).

### Genetic mechanisms of antifungal resistance

We analyzed known target genes associated with antifungal resistance in *C. glabrata* to determine the genetic basis of resistance for isolates in our collection. For this analysis, we only considered variants that were unique to antifungal-resistant *C. glabrata* isolates. We investigated genes associated with azole resistance in *C. glabrata,* including *PDR1*, *CDR1*, *PDH1* (*CDR2*), *SNQ2*, *UPC2A*, and *ERG11* (Table S4). The HS regions of *FKS1* and *FKS2* were examined in micafungin-resistant *C. glabrata*. For the single amphotericin B non-wild-type isolate in our collection, we examined *ERG2*, *ERG3*, and *ERG6*. All variants identified in the analyzed genes are described in Table S4.

#### PDR1

Azole resistance in *C. glabrata* has been previously associated with three key regions in *PDR1*: the ID (312–382 aa), MHR (539–632 aa), and TAC (800–1107 aa) (16). Thirty-six *PDR1* variants were identified in 38/52 (73.1%) fluconazole-resistant *C. glabrata* isolates (Table 3). Most *PDR1* substitutions identified in fluconazole-resistant *C. glabrata* isolates were in gene regions that have previously been associated with azole drug resistance (ID, *n* = 6; MHR, *n* = 5; and TAC, *n* = 13) (Table 3). Twelve variants were outside of the regions previously described as being associated with azole resistance, spanning amino acid positions 124–291 (region preceding the ID) and 693–772 (between the MHR and the TAC regions) (Table 3).

Only three fluconazole-resistant isolates (7.9%) in our collection had *PDR1* variants that were previously experimentally tested and shown to confer azole resistance in *C. glabrata*, namely G346D (45, 48), D876Y (16), and G1099D (45) (Table 3). The remaining 35 (92.1%) fluconazole-resistant isolates had either a suspected *PDR1* resistance variant (i.e., not experimentally confirmed) reported in at least one previous study or a potential novel *PDR1*-resistant variant reported for the first time in this study (Table 3).

Thirteen *PDR1* variants suspected to confer azole resistance in *C. glabrata* were found in 17 fluconazole-resistant isolates (Table 3). Suspected *PDR1* variants are those that were identified in only azole-resistant *C. glabrata* isolates in at least one other previous study. Most isolates harbored a single, unique suspected *PDR1* resistance variant, with the exceptions of G583S (*n* = 4) and D876N (*n* = 3), which were found in multiple isolates. Suspected *PDR1* variants K274E and D876N occupy the same positions as experimentally confirmed variants K274N (56) and D876Y (16), respectively.

Twenty potentially novel *PDR1* resistance variants were identified in 20 fluconazole-resistant *C. glabrata* isolates and were absent in susceptible-dose dependent isolates (Table 3). To our knowledge, this is the first instance these *PDR1* variants have been reported. Most isolates with novel *PDR1* variants had a single, unique variant except for N764I, which was found in two isolates. Some potential novel *PDR1* resistance variants, including W256R (57), L291F (16, 51), G611V (46), R761G (46, 54), N764I (16, 43, 49), and E1083G (16, 50), were located in the same positions as previously suspected *PDR1* resistance variants but with an alternate amino acid change.

Several *PDR1* variants were identified in our collection that did not contribute to azole resistance in *C. glabrata. PDR1* variants V27A, S391L, T488R, and S651N were present exclusively in susceptible-dose dependent isolates, while S76P, V91I, L98S, V134A, L139I, T143P, D243N, R250K, G493D, and T745A were identified in both susceptible-dose dependent and resistant isolates (Table S4).

#### Other genes associated with azole resistance

Apart from *PDR1*, there are several other genes that have been implicated in azole resistance in *C. glabrata* (Table S4). We investigated variants in efflux pumps belonging to the ABC transporter superfamily (e.g., *CDR1*, *PDH1*, and *SNQ2*) as potential mechanisms of azole resistance. There were four fluconazole-resistant *C. glabrata* isolates that had *CDR1* variants (H1098Y, *n* = 3; and A1120T, *n* = 1). *PDH1* (*CDR2*) variants were found in three fluconazole-resistant *C. glabrata* isolates (G872S, *n* = 1; H1129R, *n* = 1; and K1130I, *n* = 1). Five fluconazole-resistant isolates had variants in *SNQ2* (T613S, *n* = 2; G624R, *n* = 1; E823D, *n* = 1; and K830N, *n* = 1). Substitutions in *ERG11* and *UPC2A* are known mechanisms of azole resistance in other *Candida* species, such as *C. albicans*; however, little evidence has been reported in *C. glabrata*. Seven fluconazole-resistant *C. glabrata* isolates (L20P, *n* = 1; L97F, *n* = 1; K152R, *n* = 1; and V155A, *n* = 4) had *ERG11* variants, while two fluconazole-resistant isolates had *UPC2A* variants (T260QinsQQ, *n* = 1; and L528I, *n* = 1). Isolates that carried either *CDR1* A1120T, *SNQ2* E823D/K830N, or *ERG11* L20P did not contain *PDR1* substitutions that could potentially represent genetic determinants of azole resistance (Table S4). The *CDR1* variant was located downstream of the second ABC transporter (857-1099 aa), while the *SNQ2* variants were located upstream. There were no similar variants identified in *C. glabrata* or other *Candida* species that were associated with azole resistance in either *CDR1* or *SNQ2* (58). The *ERG11* L20P variant was located near the N-terminal, distant from the catalytic region that is associated with resistance. In *C. albicans*, an adjacent substitution in *ERG11* (Q21L) was identified but had a limited effect on azole susceptibility when experimentally tested (59). Further testing is required to experimentally confirm the role of these potential novel variants in azole resistance in *C. glabrata*.

#### *FKS* genes

Substitutions in *FKS1* (amino acid positions 625 and 629 in HS1) and *FKS2* (positions 659, 660, 663, and 664 in HS1; positions 1375 and 1377 in HS2) are most strongly associated with echinocandin resistance in *C. glabrata* (21). In our collection, there were 13 micafungin-resistant *C. glabrata* isolates, and 12 (92.3%) had *FKS* HS variants that were previously correlated with echinocandin resistance, while one isolate was wild type (Table 4). Most variants (10 out of 12) were found at positions 659 and 663 in *FKS2* HS1 (F659del, *n* = 3; S663P, *n* = 6; and S663F, *n* = 1). The remaining two variants were in *FKS1* HS1 (S629P, *n* = 1; and D632V, *n* = 1). Of the four micafungin non-susceptible *C. glabrata* isolates (MYC-19-0008, MYC-19-0272, and MYC-19-0315) that had intermediate MICs, only one isolate (MYC-19-0315) had an *FKS* variant that was previously considered to be weakly associated with echinocandin resistance (*FKS2* HS2, R1378S).

**TABLE 4:** *FKS1* and *FKS2* hotspot substitutions identified in micafungin-resistant (*n* = 13) and intermediate (*n* = 4) *C. glabrata* isolates^*a*^

Cluster	Isolate	Region	MFG MIC (µg/mL)	*FKS1* HS1^*b*^	*FKS2* HS1^*c*^	*FKS2* HS2^*d*^
I	MYC-19-0091	Central East	0.25	WT	WT	WT
	MYC-19-0093	Central East	2	WT	**S663P**	WT
	MYC-19-0094	Central East	8	WT	**S663P**	WT
	MYC-19-0095	Central East	4	WT	**F659del**	WT
	MYC-19-0386	Central East	1	WT	**S663P**	WT
III	MYC-19-0161	West	0.25	D632V	WT	WT
	MYC-19-0271	Central West	1	WT	**S663F**	WT
	MYC-19-0272^*e*^	Central West	0.12 (I)	WT	WT	WT
VIII	MYC-19-0092	Central East	4	**S629P**	WT	WT
	MYC-19-0145	Central West	1	WT	**S663P**	WT
	MYC-19-0266^*e*^	Central West	0.12 (I)	WT	WT	WT
	MYC-19-0269	Central West	0.5	WT	**F659del**	WT
XIII	MYC-19-0315^*e*^	Central East	0.12 (I)	WT	WT	R1378S
	MYC-19-0008^*e*^	East	0.12 (I)	WT	WT	WT
XIV	MYC-19-0180	West	2	WT	**S663P**	WT
XV	MYC-19-0097	Central East	2	WT	**F659del**	WT
	MYC-19-0159	West	2	WT	**S663P**	WT

^*a*^ *FKS* substitutions in bold are those documented to confer a strong resistance phenotype. MFG, micafungin; MIC, minimum inhibitory concentration; HS, hot-spot; WT, wild type.

^*b*^ *FKS1* HS1 625-FLILSLRDP.

^*c*^ *FKS2* HS1 659-FLILSLRDP.

^*d*^ *FKS2* HS2 1374-DWIRRYTL.

^*e*^ Intermediate (I) isolate.

#### *ERG* genes

The mechanisms of polyene resistance in *C. glabrata* are not well characterized; however, specific *ERG* genes, including *ERG2*, *ERG3*, and *ERG6*, involved in the ergosterol biosynthetic pathway, have been implicated (Table S4). There were no variants of interest identified in our single amphotericin B non-wild-type isolate.

#### Copy number variant analysis

We identified CNVs in genes unique to fluconazole-resistant *C. glabrata* isolates. In total, there were 146 genes with copy numbers that deviated from the expected ploidy (Table S5). One isolate (MYC-19-0345) had two copies of *PDR1* in combination with suspected (G1079R) and potential novel (G189V) *PDR1* substitutions. There were amplifications of three other genes (CAGL0D06512g, CAGL0K05841g, and CAGL0F08041g) that may be associated with azole resistance in *C. glabrata* (Table S5). Further testing is required to determine the significance of increased gene copies on azole resistance in *C. glabrata*.

### Associations between clusters and resistance variants in the Canadian collection

There were several examples of cluster-specific variants. The four isolates (MYC-19-0087, MYC-19-0181, MYC-19-0317, and MYC-19-0334; 17-70 SNVs) that had the suspected *PDR1* variant G583S all belonged to cluster I. There were also two isolates in cluster III (MYC-19-0015 and MYC-19-0083, 59 SNVs) that harbored the potential novel *PDR1* N764I variant. The three isolates (MYC-19-0015, MYC-19-0083, and MYC-19-0333; 59-300 SNVs) that harbored the *CDR1* variant H1098Y were all located in cluster III. MYC-19-0015 and MYC-19-0083 (cluster III, 59 SNVs) also contained the T613S variant in *SNQ2*. All four isolates (MYC-19-0087, MYC-19-0181, MYC-19-0317, and MYC-19-0334; 17-70 SNVs) that had the *ERG11* V155A variant were present in cluster I.

There were also examples of variants that were present in multiple clusters. The three isolates (MYC-19-0273, MYC-19-0297, and MYC-19-0320; 23338-24371 SNVs) that had the suspected *PDR1* variant D876N were present in three separate clusters. *PDR1* variants occupying the same position but with an alternate amino acid were found within the same and different clusters. The confirmed *PDR1* variant D876Y found in a single isolate (MYC-19-0319) was also in the same cluster as an isolate that carried the D876N *PDR1* variant. There were two isolates (MYC-19-0025 and MYC-19-0346) that had a *PDR1* variant in position R761 with two different amino acid changes that were found in separate clusters. Most *FKS* variants identified in more than one micafungin-resistant isolate did not group together within clusters. The six isolates (MYC-19-0093, MYC-19-0094, MYC-19-0145, MYC-19-0159, MYC-19-0180, and MYC-19-0386) that carried the *FKS2* HS1 variant S663P were found in four separate clusters (clusters I, VIII, XIV, and XV; 231-26990 SNVs). *FKS* variants that had different amino acid alterations at the same position were also not associated with the same cluster. For example, the *FKS2* HS1 variant S633F was found in a different cluster from the six isolates that contained the S663P variant.

## DISCUSSION

The global rise of invasive *C. glabrata* is worrisome since infections are becoming increasingly associated with antifungal resistance (7). While the rates of azole and echinocandin resistance in Canada were low during 2012–2016, higher rates have been reported elsewhere, highlighting the importance of understanding the mechanisms and dissemination of resistance (8, 60). In this study, we sequenced a comprehensive collection of 142 clinical *C. glabrata* isolates from across Canada, representing the largest national genomic study of *C. glabrata* from a single country to date. This collection was uniquely enriched with antifungal-resistant isolates from invasive infections to better understand the genomic phylogeny of resistant infection types. A genomics approach was also used to identify variants in genes associated with antifungal resistance to determine the genetic basis of resistance observed by phenotypic susceptibility testing.

Whole-genome sequencing is a powerful tool for resolving the genomic phylogeny of *C. glabrata*, offering high resolution and superior discriminatory power by analyzing genetic variation across the entire genome (26). The maximum-likelihood phylogeny of *C. glabrata* isolates from Canada shown in this study represented more than 75% of the core genome, capturing a far stronger and more finely resolved phylogenetic signal compared to other traditional typing methods, such as MLST, which typically rely on six genetic loci (*FKS1*, *LEU2*, *NMT1*, *TRP1*, *UGP1*, and *URA3*) to differentiate strains of *C. glabrata* (61).

A previous study based on 32 globally distributed isolates proposed the existence of seven major clades of *C. glabrata* (52). Our global analysis, which included these isolates, provided further evidence that the diversity of *C. glabrata* is much greater than originally observed, consistent with other studies (57, 62). In the absence of previously published criteria to define clusters, we used a cutoff of 1,000 SNVs to define a cluster. Clusters were separated by at least 20,000 SNVs in most cases. Using these criteria, we determined there were at least 15 genetically related lineages of *C. glabrata* in Canada. As more sequences of *C. glabrata* become publicly available for phylogenomic analysis, developing a standardized set of criteria for denoting clusters based on SNV thresholds will allow for more meaningful comparisons across studies.

*C. glabrata* has sometimes displayed geographic specificity of clusters (61). There were no significant associations between clusters and regions within Canada; however, some clusters were significantly associated with isolates from Canada, Australia, and Taiwan in our global analysis. In terms of infection source, previous studies have not found differences in clusters based on invasive and non-invasive isolates (63). Our study supports these findings as clusters were not associated with sterile or non-sterile sites of infection. We did, however, identify a large cluster that was significantly associated with antifungal resistance in our Canadian collection. Cluster I (*n* = 29) contained sterile and non-sterile isolates from all regions of Canada, with approximately two-thirds of the isolates resistant to at least one antifungal drug. This is the first reported instance of a *C. glabrata* lineage being associated with antifungal resistance to our knowledge, which may provide evidence of a strain of C*. glabrata* with a higher propensity to develop resistance.

There were several pairs of isolates in our collection that were serial isolates from the same patient. These within-patient isolates were closely related, differing by <20 SNVs, suggesting the same strain was isolated from the patient each time. One pair of isolates in our collection had different susceptibilities, the first isolate being fluconazole-resistant, while the second was fluconazole-susceptible, dose-dependent, but micafungin-resistant. This may reflect the evolution of resistance that can occur within a patient due to fitness costs and selective pressure during antifungal therapy. Other studies have included isolates from the same patient (57, 62). In one study, four pairs of isolates from separate patients were sequenced to determine if the patient harbored more than one strain of *C. glabrata*. Of the four pairs of isolates, only one patient had two different strains of *C. glabrata* (25). In one study, blood cultures from patients were observed to contain genetically diverse strains (64).

Whole-genome sequencing has also been used to detect molecular mechanisms of resistance in *C. glabrata*. We examined genes associated with azole and echinocandin resistance to determine the genetic basis of antifungal resistance. There were 58 fluconazole-resistant *C. glabrata* isolates, and 38 had variants in *PDR1* that were primarily found in regions that were previously correlated with azole resistance. Few studies have experimentally confirmed the effect of *PDR1* variants on azole resistance (14–16, 45, 56, 65). Only three isolates in our collection harbored experimentally verified variants of *PDR1*. Over 150 *PDR1* variants identified in previous studies of *C. glabrata* are suspected to decrease azole susceptibility; however, these variants have not been experimentally validated (16, 42–44, 46, 47, 49–51, 57, 66–68). Most isolates in our collection had *PDR1* variants that could potentially describe azole resistance. Some *PDR1* variants have been previously reported in azole-resistant *C. glabrata* isolates in at least one other study, and detection of these suspected *PDR1* variants in our collection lends further support that these variants may indeed be associated with azole resistance. Some suspected *PDR1* variants fall outside of *PDR1* regions associated with azole resistance. Twenty *PDR1* variants were reported for the first time here. Some variants occupied the same position as previously identified *PDR1* variants, but with alternate amino acid substitutions. Experimental testing of a greater number of *PDR1* variants identified within and outside of the key regions will better delineate variants associated with azole resistance.

Almost all micafungin-resistant isolates in our collection had *FKS1* or *FKS2* variants (at position 629/632 or 659/663, respectively) that are strongly associated with echinocandin resistance. A single isolate harboring no *FKS* mutations yet was phenotypically resistant, suggesting that it may have a currently unknown mechanism of resistance. Our study also supports the previous finding that echinocandin resistance in *C. glabrata* is more strongly associated with *FKS2* HS variants compared to *FKS1* in 10 out of 13 variants we have described (46, 69).

Our study had several limitations. The scope was limited to the analysis of genes known to be associated with resistance to the three major classes of antifungals and provides no insight into novel resistance genes. Future genome-wide association studies may identify significant variants in novel resistance genes or the combined effect of mutations in multiple target genes. Since we did not have access to patient health information, we were unable to analyze correlations between patient treatment history and antifungal resistance. Similarly, without access to travel history, we were also unable to determine if the Canadian isolates clustered within the global analysis were potentially acquired during travel or medical tourism. Finally, experimentally validating the role of suspected and potential novel resistance variants through gene editing and/or gene expression analysis is essential to confirm their role in antifungal resistance.

In summary, whole-genome sequencing of the largest collection of clinical *C. glabrata* isolates from Canada to date was useful for determining the genomic phylogeny and potential genetic mechanisms of antifungal resistance of this pathogen across Canada. We observed high levels of genetic diversity, with at least one cluster associated with phenotypic antifungal resistance. Most fluconazole-resistant isolates harbored suspected or potential novel resistance variants in *PDR1*, while micafungin resistance was primarily described by substitutions in the HS regions of *FKS* genes. Antifungal-resistant isolates were present in almost all clusters, suggesting that resistance most likely arises independently from selective pressure during antifungal therapy rather than dissemination of resistant clones. Our study provides a baseline for future genomic studies of *C. glabrata* and outbreak investigations in Canada. Future work should focus on experimentally testing resistance variants to improve genotypic prediction of antifungal resistance in *C. glabrata*.

## Data Availability

Sequence data for all isolates were deposited to NCBI SRA under BioProject PRJNA592373.

## SUPPLEMENTAL MATERIAL

Supplemental tables spectrum.01803-25-s0001.docx Tables S1 to S5.

ASM does not own the copyrights to Supplemental Material that may be linked to, or accessed through, an article. The authors have granted ASM a non-exclusive, world-wide license to publish the Supplemental Material files. Please contact the corresponding author directly for reuse.

## References

[1] Nucci M, Anaissie E. 2001. Revisiting the source of candidemia: skin or gut? Clin Infect Dis 33:1959–1967. doi:10.1086/323759 11702290

[2] Rodrigues CF, Silva S, Henriques M. 2014. Candida glabrata: a review of its features and resistance. Eur J Clin Microbiol Infect Dis 33:673–688. doi:10.1007/s10096-013-2009-3 24249283

[3] Vazquez JA, Dembry LM, Sanchez V, Vazquez MA, Sobel JD, Dmuchowski C, Zervos MJ. 1998. Nosocomial Candida glabrata colonization: an epidemiologic study. J Clin Microbiol 36:421–426. doi:10.1128/JCM.36.2.421-426.1998 9466752 PMC104553

[4] Goemaere B, Lagrou K, Spriet I, Hendrickx M, Becker P. 2018. Clonal spread of Candida glabrata bloodstream isolates and fluconazole resistance affected by prolonged exposure: a 12-year single-center study in Belgium. Antimicrob Agents Chemother 62:e00591-18. doi:10.1128/AAC.00591-18 29784839 PMC6105788

[5] Abbes S, Sellami H, Sellami A, Makni F, Mahfoudh N, Makni H, Khaled S, Ayadi A. 2011. Microsatellite analysis and susceptibility to FCZ of Candida glabrata invasive isolates in Sfax Hospital, Tunisia. Med Mycol 49:10–15. doi:10.3109/13693786.2010.493561 20586679

[6] Arastehfar A, Lass-Flörl C, Garcia-Rubio R, Daneshnia F, Ilkit M, Boekhout T, Gabaldon T, Perlin DS. 2020. The quiet and underappreciated rise of drug-resistant invasive fungal pathogens. J Fungi 6:138. doi:10.3390/jof6030138 PMC7557958 32824785

[7] Pfaller MA, Diekema DJ, Turnidge JD, Castanheira M, Jones RN. 2019. Twenty years of the SENTRY antifungal surveillance program: results for Candida species From 1997-2016. Open Forum Infect Dis 6:S79–S94. doi:10.1093/ofid/ofy358 30895218 PMC6419901

[8] Fuller J, Dingle TC, Bull A, Shokoples S, Laverdière M, Baxter MR, Adam HJ, Karlowsky JA, Zhanel GG, Canadian Antimicrobial Resistance Alliance (CARA) and CANWARD. 2019. Species distribution and antifungal susceptibility of invasive Candida isolates from Canadian hospitals: results of the CANWARD 2011-16 study. J Antimicrob Chemother 74:iv48–iv54. doi:10.1093/jac/dkz287 31505645

[9] Chapman B, Slavin M, Marriott D, et al.. 2017. Changing epidemiology of candidaemia in Australia. J Antimicrob Chemother 72:1103–1108. doi:10.1093/jac/dkw422 28364558

[10] Astvad KMT, Johansen HK, Røder BL, Rosenvinge FS, Knudsen JD, Lemming L, Schønheyder HC, Hare RK, Kristensen L, Nielsen L, Gertsen JB, Dzajic E, Pedersen M, Østergård C, Olesen B, Søndergaard TS, Arendrup MC. 2018. Update from a 12-year nationwide fungemia surveillance: increasing intrinsic and acquired resistance causes concern. J Clin Microbiol 56:e01564-17. doi:10.1128/JCM.01564-17 29212705 PMC5869841

[11] Tsay SV, Mu Y, Williams S, Epson E, Nadle J, Bamberg WM, Barter DM, Johnston HL, Farley MM, Harb S, et al.. 2020. Burden of candidemia in the United States, 2017. Clin Infect Dis 71:e449–e453. doi:10.1093/cid/ciaa193 32107534

[12] Aldejohann AM, Herz M, Martin R, Walther G, Kurzai O. 2021. Emergence of resistant Candida glabrata in Germany. JAC Antimicrob Resist 3:dlab122. doi:10.1093/jacamr/dlab122 34377983 PMC8346698

[13] Arendrup MC, Patterson TF. 2017. Multidrug-resistant Candida: epidemiology, molecular mechanisms, and treatment. J Infect Dis 216:S445–S451. doi:10.1093/infdis/jix131 28911043

[14] Tsai HF, Krol AA, Sarti KE, Bennett JE. 2006. Candida glabrata PDR1, a transcriptional regulator of a pleiotropic drug resistance network, mediates azole resistance in clinical isolates and petite mutants. Antimicrob Agents Chemother 50:1384–1392. doi:10.1128/AAC.50.4.1384-1392.2006 16569856 PMC1426987

[15] Vermitsky J-P, Earhart KD, Smith WL, Homayouni R, Edlind TD, Rogers PD. 2006. Pdr1 regulates multidrug resistance in Candida glabrata: gene disruption and genome-wide expression studies. Mol Microbiol 61:704–722. doi:10.1111/j.1365-2958.2006.05235.x 16803598

[16] Ferrari S, Ischer F, Calabrese D, Posteraro B, Sanguinetti M, Fadda G, Rohde B, Bauser C, Bader O, Sanglard D. 2009. Gain of function mutations in CgPDR1 of Candida glabrata not only mediate antifungal resistance but also enhance virulence. PLoS Pathog 5:e1000268. doi:10.1371/journal.ppat.1000268 19148266 PMC2607542

[17] Poláková S, Blume C, Zárate JA, Mentel M, Jørck-Ramberg D, Stenderup J, Piskur J. 2009. Formation of new chromosomes as a virulence mechanism in yeast Candida glabrata. Proc Natl Acad Sci USA 106:2688–2693. doi:10.1073/pnas.0809793106 19204294 PMC2637908

[18] Peng Y, Dong D, Jiang C, Yu B, Wang X, Ji Y. 2012. Relationship between respiration deficiency and azole resistance in clinical Candida glabrata. FEMS Yeast Res 12:719–727. doi:10.1111/j.1567-1364.2012.00821.x 22713096

[19] Zheng Q, Liu J, Qin J, Wang B, Bing J, Du H, Li M, Yu F, Huang G. 2022. Ploidy variation and spontaneous haploid-diploid switching of Candida glabrata clinical isolates. mSphere 7:e00260-22. doi:10.1128/msphere.00260-22 35727043 PMC9429935

[20] Whaley SG, Rogers PD, Rogers PD. 2016. Azole resistance in Candida glabrata. Curr Infect Dis Rep 18:41. doi:10.1007/s11908-016-0554-5 27761779

[21] Morio F, Jensen RH, Le Pape P, Arendrup MC. 2017. Molecular basis of antifungal drug resistance in yeasts. Int J Antimicrob Agents 50:599–606. doi:10.1016/j.ijantimicag.2017.05.012 28669835

[22] Hull CM, Parker JE, Bader O, Weig M, Gross U, Warrilow AGS, Kelly DE, Kelly SL. 2012. Facultative sterol uptake in an ergosterol-deficient clinical isolate of Candida glabrata harboring a missense mutation in ERG11 and exhibiting cross-resistance to azoles and amphotericin B. Antimicrob Agents Chemother 56:4223–4232. doi:10.1128/AAC.06253-11 22615281 PMC3421581

[23] Hull CM, Bader O, Parker JE, Weig M, Gross U, Warrilow AGS, Kelly DE, Kelly SL. 2012. Two clinical isolates of Candida glabrata exhibiting reduced sensitivity to amphotericin B both harbor mutations in ERG2. Antimicrob Agents Chemother 56:6417–6421. doi:10.1128/AAC.01145-12 23027188 PMC3497184

[24] Geber A, Hitchcock CA, Swartz JE, Pullen FS, Marsden KE, Kwon-Chung KJ, Bennett JE. 1995. Deletion of the Candida glabrata ERG3 and ERG11 genes: effect on cell viability, cell growth, sterol composition, and antifungal susceptibility. Antimicrob Agents Chemother 39:2708–2717. doi:10.1128/AAC.39.12.2708 8593007 PMC163017

[25] McTaggart LR, Cabrera A, Cronin K, Kus JV. 2020. Antifungal susceptibility of clinical yeast isolates from a large Canadian reference laboratory and application of whole-genome sequence analysis to elucidate mechanisms of acquired resistance. Antimicrob Agents Chemother 64:e00402-20. doi:10.1128/AAC.00402-20 32571812 PMC7449159

[26] Gabaldón T, Gómez-Molero E, Bader O. 2020. Molecular typing of Candida glabrata. Mycopathologia 185:755–764. doi:10.1007/s11046-019-00388-x 31617105

[27] CLSI. 2017. Reference method for broth dilution antifungal susceptibility testing of yeasts. In CLSI Standard M27, 4th ed. CLSI, Wayne, PA.

[28] CLSI. 2022. Performance standards for antifungal susceptibility testing of yeasts. In CLSI supplement M27M44S, 3rd ed. CLSI, Wayne, PA.

[29] CLSI. 2022. Epidemiological cutoff values for antifungal susceptibility testing. In CLSI supplement M57S, 4th ed. CLSI, Wayne, PA.

[30] Chen S, Zhou Y, Chen Y, Gu J. 2018. Fastp: an ultra-fast all-in-one FASTQ preprocessor. Bioinformatics 34:i884–i890. doi:10.1093/bioinformatics/bty560 30423086 PMC6129281

[31] Petkau A, Mabon P, Sieffert C, Knox NC, Cabral J, Iskander M, Iskander M, Weedmark K, Zaheer R, Katz LS, Nadon C, Reimer A, Taboada E, Beiko RG, Hsiao W, Brinkman F, Graham M, Van Domselaar G. 2017. SNVPhyl: a single nucleotide variant phylogenomics pipeline for microbial genomic epidemiology. Microb Genom 3:e000116. doi:10.1099/mgen.0.000116 29026651 PMC5628696

[32] Skrzypek MS, Binkley J, Binkley G, Miyasato SR, Simison M, Sherlock G. 2017. The Candida Genome Database (CGD): incorporation of Assembly 22, systematic identifiers and visualization of high throughput sequencing data. Nucleic Acids Res 45:D592–D596. doi:10.1093/nar/gkw924 27738138 PMC5210628

[33] Letunic I, Bork P. 2024. Interactive Tree of Life (iTOL) v6: recent updates to the phylogenetic tree display and annotation tool. Nucleic Acids Res 52:W78–W82. doi:10.1093/nar/gkae268 38613393 PMC11223838

[34] Li H. 2013. Aligning sequence reads, clone sequences and assembly contigs with BWA-MEM. arXiv. doi:10.48550/arXiv.1303.3997

[35] Danecek P, Bonfield JK, Liddle J, Marshall J, Ohan V, Pollard MO, Whitwham A, Keane T, McCarthy SA, Davies RM, Li H. 2021. Twelve years of SAMtools and BCFtools. Gigascience 10:giab008. doi:10.1093/gigascience/giab008 33590861 PMC7931819

[36] Garrison E, Marth G. 2012. Haplotype-based variant detection from short-read sequencing. arXiv. doi:10.48550/arXiv.1207.3907

[37] Cingolani P, Platts A, Wang L, et al.. 2012. A program for annotating and predicting the effects of single nucleotide polymorphisms, SnpEff: SNPs in the genome of Drosophila melanogaster strain w1118; iso-2; iso-3. Fly (Austin) 6:80–92. doi:10.4161/fly.19695 22728672 PMC3679285

[38] Cingolani P, Patel VM, Coon M, Nguyen T, Land SJ, Ruden DM, Lu X. 2012. Using Drosophila melanogaster as a model for genotoxic chemical mutational studies with a new program, SnpSift. Front Genet 3:35. doi:10.3389/fgene.2012.00035 22435069 PMC3304048

[39] Ramírez F, Dündar F, Diehl S, Grüning BA, Manke T. 2014. deepTools: a flexible platform for exploring deep-sequencing data. Nucleic Acids Res 42:W187–91. doi:10.1093/nar/gku365 24799436 PMC4086134

[40] Quinlan AR, Hall IM. 2010. BEDTools: a flexible suite of utilities for comparing genomic features. Bioinformatics 26:841–842. doi:10.1093/bioinformatics/btq033 20110278 PMC2832824

[41] RStudio Team. 2025. RStudio: integrated development environment for R. Available from: http://www.rstudio.com

[42] Healey KR, Zhao Y, Perez WB, Lockhart SR, Sobel JD, Farmakiotis D, Kontoyiannis DP, Sanglard D, Taj-Aldeen SJ, Alexander BD, Jimenez-Ortigosa C, Shor E, Perlin DS. 2016. Prevalent mutator genotype identified in fungal pathogen Candida glabrata promotes multi-drug resistance. Nat Commun 7:11128. doi:10.1038/ncomms11128 27020939 PMC5603725

[43] Won EJ, Choi MJ, Kim M-N, Yong D, Lee WG, Uh Y, Kim TS, Byeon SA, Lee SY, Kim SH, Shin JH. 2021. Fluconazole-resistant Candida glabrata bloodstream isolates, South Korea, 2008-2018. Emerg Infect Dis 27:779–788. doi:10.3201/eid2703.203482 33624581 PMC7920659

[44] Lim HJ, Shin JH, Kim M-N, Yong D, Byun SA, Choi MJ, Lee SY, Won EJ, Kee S-J, Kim SH, Shin M-G. 2020. Evaluation of two commercial broth microdilution methods using different interpretive criteria for the detection of molecular mechanisms of acquired azole and echinocandin resistance in four common Candida species. Antimicrob Agents Chemother 64:e00740-20. doi:10.1128/AAC.00740-20 32900684 PMC7577149

[45] Ni Q, Wang C, Tian Y, Dong D, Jiang C, Mao E, Peng Y. 2018. CgPDR1 gain-of-function mutations lead to azole-resistance and increased adhesion in clinical Candida glabrata strains. Mycoses 61:430–440. doi:10.1111/myc.12756 29464833

[46] Castanheira M, Deshpande LM, Davis AP, Carvalhaes CG, Pfaller MA. 2022. Azole resistance in Candida glabrata clinical isolates from global surveillance is associated with efflux overexpression. J Glob Antimicrob Resist 29:371–377. doi:10.1016/j.jgar.2022.05.004 35577042

[47] Hou X, Xiao M, Wang H. 2018. Profiling of PDR1 and MSH2 in Candida glabrata bloodstream isolates from a multicenter study in China. Antimicrob Agents Chemother 62:e00153–18. doi:10.1128/AAC.00153-18 29581110 PMC5971605

[48] Tian Y, Zhuang Y, Chen Z, Mao Y, Zhang J, Lu R, Guo L. 2020. A gain-of-function mutation in PDR1 of Candida glabrata decreases EPA1 expression and attenuates adherence to epithelial cells through enhancing recruitment of the mediator subunit Gal11A. Microbiol Res 239:126519. doi:10.1016/j.micres.2020.126519 32563123

[49] Yao D, Chen J, Chen W, Li Z, Hu X. 2019. Mechanisms of azole resistance in clinical isolates of Candida glabrata from two hospitals in China. Infect Drug Resist 12:771–781. doi:10.2147/IDR.S202058 31118695 PMC6498982

[50] Katiyar S, Shiffrin E, Shelton C, Healey K, Vermitsky JP, Edlind T. 2016. Evaluation of polymorphic locus sequence typing for Candida glabrata epidemiology. J Clin Microbiol 54:1042–1050. doi:10.1128/JCM.03106-15 26842706 PMC4809956

[51] Spettel K, Barousch W, Makristathis A, Zeller I, Nehr M, Selitsch B, Lackner M, Rath P-M, Steinmann J, Willinger B. 2019. Analysis of antifungal resistance genes in Candida albicans and Candida glabrata using next generation sequencing. PLoS One 14:e0210397. doi:10.1371/journal.pone.0210397 30629653 PMC6328131

[52] Carreté L, Ksiezopolska E, Pegueroles C, Gómez-Molero E, Saus E, Iraola-Guzmán S, Loska D, Bader O, Fairhead C, Gabaldón T. 2018. Patterns of genomic variation in the opportunistic pathogen Candida glabrata suggest the existence of mating and a secondary association with humans. Curr Biol 28:15–27. doi:10.1016/j.cub.2017.11.027 29249661 PMC5772174

[53] Szarvas J, Rebelo AR, Bortolaia V, Leekitcharoenphon P, Schrøder Hansen D, Nielsen HL, Nørskov-Lauritsen N, Kemp M, Røder BL, Frimodt-Møller N, Søndergaard TS, Coia JE, Østergaard C, Westh H, Aarestrup FM. 2021. Danish whole-genome-sequenced Candida albicans and Candida glabrata samples fit into globally prevalent clades. J Fungi 7:962. doi:10.3390/jof7110962 PMC8622182 34829249

[54] Barber AE, Weber M, Kaerger K, Linde J, Gölz H, Duerschmied D, Markert A, Guthke R, Walther G, Kurzai O. 2019. Comparative genomics of serial Candida glabrata isolates and the rapid acquisition of echinocandin resistance during therapy. Antimicrob Agents Chemother 63:e01628-18. doi:10.1128/AAC.01628-18 30478162 PMC6355595

[55] Vale-Silva L, Beaudoing E, Tran VDT, Sanglard D. 2017. Comparative genomics of two sequential Candida glabrata clinical isolates. G3 (Bethesda) 7:2413–2426. doi:10.1534/g3.117.042887 28663342 PMC5555451

[56] Caudle KE, Barker KS, Wiederhold NP, Xu L, Homayouni R, Rogers PD. 2011. Genomewide expression profile analysis of the Candida glabrata Pdr1 regulon. Eukaryot Cell 10:373–383. doi:10.1128/EC.00073-10 21193550 PMC3067479

[57] Biswas C, Marcelino VR, Van Hal S, Halliday C, Martinez E, Wang Q, Kidd S, Kennedy K, Marriott D, Morrissey CO, Arthur I, Weeks K, Slavin MA, Sorrell TC, Sintchenko V, Meyer W, Chen SC-A. 2018. Whole genome sequencing of Australian Candida glabrata isolates reveals genetic diversity and novel sequence types. Front Microbiol 9:2946. doi:10.3389/fmicb.2018.02946 30559734 PMC6287553

[58] Bédard C, Pageau A, Fijarczyk A, Mendoza-Salido D, Alcañiz AJ, Després PC, Durand R, Plante S, Alexander EMM, Rouleau FD, et al.. 2025. FungAMR: a comprehensive database for investigating fungal mutations associated with antimicrobial resistance. Nat Microbiol 10:2338–2352. doi:10.1038/s41564-025-02084-7 40790106

[59] Flowers SA, Colón B, Whaley SG, Schuler MA, Rogers PD. 2015. Contribution of clinically derived mutations in ERG11 to azole resistance in Candida albicans. Antimicrob Agents Chemother 59:450–460. doi:10.1128/AAC.03470-14 25385095 PMC4291385

[60] Lee Y, Puumala E, Robbins N, Cowen LE. 2021. Antifungal drug resistance: molecular mechanisms in Candida albicans and beyond. Chem Rev 121:3390–3411. doi:10.1021/acs.chemrev.0c00199 32441527 PMC8519031

[61] Dodgson AR, Pujol C, Denning DW, Soll DR, Fox AJ. 2003. Multilocus sequence typing of Candida glabrata reveals geographically enriched clades. J Clin Microbiol 41:5709–5717. doi:10.1128/JCM.41.12.5709-5717.2003 14662965 PMC309006

[62] Helmstetter N, Chybowska AD, Delaney C, Da Silva Dantas A, Gifford H, Wacker T, Munro C, Warris A, Jones B, Cuomo CA, Wilson D, Ramage G, Farrer RA. 2022. Population genetics and microevolution of clinical Candida glabrata reveals recombinant sequence types and hyper-variation within mitochondrial genomes, virulence genes, and drug targets. Genetics 221:iyac031. doi:10.1093/genetics/iyac031 35199143 PMC9071574

[63] Lott TJ, Frade JP, Lyon GM, Iqbal N, Lockhart SR. 2012. Bloodstream and non-invasive isolates of Candida glabrata have similar population structures and fluconazole susceptibilities. Med Mycol 50:136–142. doi:10.3109/13693786.2011.592153 21838617

[64] Badrane H, Cheng S, Dupont CL, Hao B, Driscoll E, Morder K, Liu G, Newbrough A, Fleres G, Kaul D, Espinoza JL, Clancy CJ, Nguyen MH. 2023. Genotypic diversity and unrecognized antifungal resistance among populations of Candida glabrata from positive blood cultures. Nat Commun 14:5918. doi:10.1038/s41467-023-41509-x 37739935 PMC10516878

[65] Berila N, Subik J. 2010. Molecular analysis of Candida glabrata clinical isolates. Mycopathologia 170:99–105. doi:10.1007/s11046-010-9298-1 20232155

[66] Salazar SB, Pinheiro MJF, Sotti-Novais D, Soares AR, Lopes MM, Ferreira T, Rodrigues V, Fernandes F, Mira NP. 2022. Disclosing azole resistance mechanisms in resistant Candida glabrata strains encoding wild-type or gain-of-function CgPDR1 alleles through comparative genomics and transcriptomics. G3 (Bethesda) 12:jkac110. doi:10.1093/g3journal/jkac110 35532173 PMC9258547

[67] Tantivitayakul P, Lapirattanakul J, Kaypetch R, Muadcheingka T. 2019. Missense mutation in CgPDR1 regulator associated with azole-resistant Candida glabrata recovered from thai oral candidiasis patients. J Glob Antimicrob Resist 17:221–226. doi:10.1016/j.jgar.2019.01.006 30658200

[68] Khalifa HO, Arai T, Majima H, Watanabe A, Kamei K. 2020. Genetic basis of azole and echinocandin resistance in clinical Candida glabrata in Japan. Antimicrob Agents Chemother 64:e00783-20. doi:10.1128/AAC.00783-20 32571826 PMC7449192

[69] Zimbeck AJ, Iqbal N, Ahlquist AM, Farley MM, Harrison LH, Chiller T, Lockhart SR. 2010. FKS mutations and elevated echinocandin MIC values among Candida glabrata isolates from U.S. population-based surveillance. Antimicrob Agents Chemother 54:5042–5047. doi:10.1128/AAC.00836-10 20837754 PMC2981257

