# Supplementary material for: Phylogenomic analysis and genetic mechanisms of antifungal resistance in clinical isolates of Candida glabrata (Nakaseomyces glabratus) from across Canada, 2013–2020
Source: Microbiol Spectr. 2025 Nov 26;14(1):e01803-25. doi: 10.1128/spectrum.01803-25 (PMC12772373; doi:10.1128/spectrum.01803-25)
Supplement: Supplemental tables — Tables S1 to S5. [file spectrum.01803-25-s0001.docx]

**Supplementary Material**

**Supplementary Table S1. Line list of *Candida glabrata* isolates (n = 142) included in this study.**

| **Isolate ID** | **Global ID** | **BioSample ID** | **Region of isolation** | **Year** | **Site of Isolation** | **FLZ (**𝝁**g/mL)** | **FLZ INT** | **MFG (**𝝁**g/mL)** | **MFG INT** | **AMB (**𝝁**g/mL)** | **AMB INT** |
| --- | --- | --- | --- | --- | --- | --- | --- | --- | --- | --- | --- |
| MYC-19-0003 | CAD1 | SAMN51609364 | East | 2018 | Blood | 4 | Susceptible dose | 0.008 | Susceptible | 1 | Wildtype |
| MYC-19-0004 | CAD2 | SAMN51609365 | East | 2018 | Blood | 32 | Susceptible dose | 0.03 | Susceptible | 1 | Wildtype |
| MYC-19-0007 | CAD3 | SAMN51609366 | East | 2018 | Blood | 8 | Susceptible dose | 0.03 | Susceptible | 1 | Wildtype |
| MYC-19-0008 | CAD4 | SAMN51609367 | East | 2018 | Blood | 64 | Resistant | 0.12 | Intermediate | 2 | Wildtype |
| MYC-19-0014 | CAD5 | SAMN51609368 | East | 2019 | Carotid tissue | 8 | Susceptible dose | 0.008 | Susceptible | 0.5 | Wildtype |
| MYC-19-0015 | CAD6 | SAMN51609369 | East | 2019 | Kidney fluid | 64 | Resistant | 0.008 | Susceptible | 0.5 | Wildtype |
| MYC-19-0016 | CAD7 | SAMN51609370 | East | 2019 | Abscess fluid | 8 | Susceptible dose | 0.015 | Susceptible | 1 | Wildtype |
| MYC-19-0017 | CAD8 | SAMN51609371 | East | 2018 | Abscess fluid | 8 | Susceptible dose | 0.008 | Susceptible | 1 | Wildtype |
| MYC-19-0018 | CAD9 | SAMN51609372 | East | 2018 | Blood | 4 | Susceptible dose | 0.008 | Susceptible | 1 | Wildtype |
| MYC-19-0024 | CAD10 | SAMN51609373 | East | 2019 | Urine - cystoscopy | 8 | Susceptible dose | 0.008 | Susceptible | 1 | Wildtype |
| MYC-19-0025 | CAD11 | SAMN51609374 | East | 2019 | Urine - cystoscopy | 64 | Resistant | 0.015 | Susceptible | 0.25 | Wildtype |
| MYC-19-0026 | CAD12 | SAMN51609375 | East | 2019 | Urine - midstream | 16 | Susceptible dose | ≤ 0.008 | Susceptible | 0.5 | Wildtype |
| MYC-19-0027 | CAD13 | SAMN51609376 | East | 2019 | Blood | 4 | Susceptible dose | 0.015 | Susceptible | 1 | Wildtype |
| MYC-19-0044 | CAD14 | SAMN51609377 | Central East | 2019 | Blood | 1 | Susceptible dose | ≤ 0.008 | Susceptible | 0.5 | Wildtype |
| MYC-19-0045 | CAD15 | SAMN51609378 | Central East | 2019 | Liquid | 1 | Susceptible dose | 0.015 | Susceptible | 0.5 | Wildtype |
| MYC-19-0046 | CAD16 | SAMN51609379 | Central East | 2019 | Liver abscess | 4 | Susceptible dose | ≤ 0.008 | Susceptible | 0.5 | Wildtype |
| MYC-19-0047 | CAD17 | SAMN51609380 | Central East | 2019 | Blood | 16 | Susceptible dose | ≤ 0.008 | Susceptible | 0.5 | Wildtype |
| MYC-19-0048 | CAD18 | SAMN51609381 | Central East | 2019 | Pus-abscess | 8 | Susceptible dose | ≤ 0.008 | Susceptible | 0.5 | Wildtype |
| MYC-19-0050 | CAD19 | SAMN51609382 | Central East | 2019 | Synovial liquid | 4 | Susceptible dose | ≤ 0.008 | Susceptible | 0.5 | Wildtype |
| MYC-19-0051 | CAD20 | SAMN51609383 | Central East | 2019 | Cyst | 8 | Susceptible dose | ≤ 0.008 | Susceptible | 0.5 | Wildtype |
| MYC-19-0052 | CAD21 | SAMN51609384 | East | 2019 | Blood | 8 | Susceptible dose | 0.015 | Susceptible | 0.5 | Wildtype |
| MYC-19-0053 | CAD22 | SAMN51609385 | East | 2019 | Blood | 8 | Susceptible dose | ≤ 0.008 | Susceptible | 0.5 | Wildtype |
| MYC-19-0082 | CAD23 | SAMN51609386 | Central East | 2018 | Blood | 128 | Resistant | 0.015 | Susceptible | 0.25 | Wildtype |
| MYC-19-0083 | CAD24 | SAMN51609387 | Central East | 2018 | Blood | 128 | Resistant | 0.015 | Susceptible | 1 | Wildtype |
| MYC-19-0084 | CAD25 | SAMN51609388 | Central East | 2018 | Blood | 8 | Susceptible dose | 0.008 | Susceptible | 1 | Wildtype |
| MYC-19-0087 | CAD26 | SAMN51609389 | Central East | 2019 | Blood | 128 | Resistant | 0.015 | Susceptible | 0.5 | Wildtype |
| MYC-19-0088 | CAD27 | SAMN51609390 | Central East | 2019 | Blood | 128 | Resistant | 0.015 | Susceptible | 1 | Wildtype |
| MYC-19-0089 | CAD28 | SAMN51609391 | Central East | 2018 | Blood | 256 | Resistant | 0.015 | Susceptible | 0.5 | Wildtype |
| MYC-19-0091 | CAD29 | SAMN51609392 | Central East | 2013 | Blood | 8 | Susceptible dose | 0.25 | Resistant | 2 | Wildtype |
| MYC-19-0092 | CAD30 | SAMN51609393 | Central East | 2014 | Blood | ≥ 256 | Resistant | 4 | Resistant | 0.5 | Wildtype |
| MYC-19-0093 | CAD31 | SAMN51609394 | Central East | 2017 | Blood | 128 | Resistant | 2 | Resistant | 1 | Wildtype |
| MYC-19-0094 | CAD32 | SAMN51609395 | Central East | 2017 | Blood | 256 | Resistant | 8 | Resistant | 1 | Wildtype |
| MYC-19-0095 | CAD33 | SAMN51609396 | Central East | 2016 | Blood | 8 | Susceptible dose | 4 | Resistant | 0.5 | Wildtype |
| MYC-19-0096 | CAD34 | SAMN51609397 | Central East | 2017 | Blood | 2 | Susceptible dose | 0.008 | Susceptible | 0.5 | Wildtype |
| MYC-19-0097 | CAD35 | SAMN51609398 | Central East | 2019 | Blood | 8 | Susceptible dose | 2 | Resistant | 0.25 | Wildtype |
| MYC-19-0098 | CAD36 | SAMN51609399 | Central East | 2016 | Blood | 32 | Susceptible dose | 0.015 | Susceptible | 2 | Wildtype |
| MYC-19-0099 | CAD37 | SAMN51609400 | Central East | 2017 | Blood | 2 | Susceptible dose | 0.015 | Susceptible | 2 | Wildtype |
| MYC-19-0100 | CAD38 | SAMN51609401 | Central East | 2018 | Blood | 16 | Susceptible dose | 0.015 | Susceptible | 2 | Wildtype |
| MYC-19-0101 | CAD39 | SAMN51609402 | Central East | 2018 | Blood | 16 | Susceptible dose | 0.015 | Susceptible | 2 | Wildtype |
| MYC-19-0102 | CAD40 | SAMN51609403 | Central East | 2018 | Blood | 8 | Susceptible dose | 0.015 | Susceptible | 0.5 | Wildtype |
| MYC-19-0103 | CAD41 | SAMN51609404 | Central East | 2018 | Blood | 16 | Susceptible dose | 0.015 | Susceptible | 1 | Wildtype |
| MYC-19-0104 | CAD42 | SAMN51609405 | Central East | 2018 | Blood | 16 | Susceptible dose | 0.008 | Susceptible | 1 | Wildtype |
| MYC-19-0105 | CAD43 | SAMN51609406 | Central East | 2019 | Blood | 8 | Susceptible dose | 0.015 | Susceptible | 0.5 | Wildtype |
| MYC-19-0106 | CAD44 | SAMN51609407 | Central East | 2019 | Blood | 8 | Susceptible dose | 0.008 | Susceptible | 0.5 | Wildtype |
| MYC-19-0107 | CAD45 | SAMN51609408 | Central East | 2019 | Blood | 8 | Susceptible dose | 0.015 | Susceptible | 0.5 | Wildtype |
| MYC-19-0108 | CAD46 | SAMN51609409 | Central East | 2019 | Blood | 16 | Susceptible dose | 0.015 | Susceptible | 0.5 | Wildtype |
| MYC-19-0109 | CAD47 | SAMN51609410 | Central East | 2019 | Blood | 16 | Susceptible dose | 0.015 | Susceptible | 0.5 | Wildtype |
| MYC-19-0145 | CAD48 | SAMN51609411 | Central West | 2017 | Blood | 0.5 | Susceptible dose | 1 | Resistant | 0.5 | Wildtype |
| MYC-19-0149 | CAD49 | SAMN51609412 | Central West | 2017 | Vagina | 64 | Resistant | 0.03 | Susceptible | 1 | Wildtype |
| MYC-19-0159 | CAD50 | SAMN51609413 | West | 2017 | Tissue - peritoneum | 4 | Susceptible dose | 2 | Resistant | 1 | Wildtype |
| MYC-19-0161 | CAD51 | SAMN51609414 | West | 2017 | Swab - mouth | > 128 | Resistant | 0.25 | Resistant | 0.25 | Wildtype |
| MYC-19-0164 | CAD52 | SAMN51609415 | Central West | 2018 | Isolate - other | 4 | Susceptible dose | ≤ 0.008 | Susceptible | 2 | Wildtype |
| MYC-19-0166 | CAD53 | SAMN51609416 | Central West | 2018 | Blood | 128 | Resistant | 0.015 | Susceptible | 0.5 | Wildtype |
| MYC-19-0167 | CAD54 | SAMN51609417 | Central West | 2018 | Blood | > 128 | Resistant | 0.03 | Susceptible | 0.5 | Wildtype |
| MYC-19-0168 | CAD55 | SAMN51609418 | West | 2018 | Aspirate (sterile) - pancreas | 16 | Susceptible dose | ≤ 0.008 | Susceptible | 1 | Wildtype |
| MYC-19-0172 | CAD56 | SAMN51609419 | West | 2018 | Blood | 8 | Susceptible dose | ≤ 0.008 | Susceptible | 2 | Wildtype |
| MYC-19-0173 | CAD57 | SAMN51609420 | West | 2018 | Swab - sternum | 8 | Susceptible dose | 0.015 | Susceptible | 2 | Wildtype |
| MYC-19-0174 | CAD58 | SAMN51609421 | West | 2018 | Blood | 64 | Resistant | ≤ 0.008 | Susceptible | 1 | Wildtype |
| MYC-19-0180 | CAD59 | SAMN51609422 | West | 2018 | Blood | 8 | Susceptible dose | 2 | Resistant | 0.5 | Wildtype |
| MYC-19-0181 | CAD60 | SAMN51609423 | West | 2018 | Pelvis | 128 | Resistant | ≤ 0.008 | Susceptible | 0.5 | Wildtype |
| MYC-19-0184 | CAD61 | SAMN51609424 | Central West | 2018 | Abdomen | 4 | Susceptible dose | ≤ 0.008 | Susceptible | 0.5 | Wildtype |
| MYC-19-0185 | CAD62 | SAMN51609425 | Central West | 2018 | Peritoneal fluid | 8 | Susceptible dose | 0.03 | Susceptible | 1 | Wildtype |
| MYC-19-0186 | CAD63 | SAMN51609426 | Central West | 2018 | Bile | 2 | Susceptible dose | 0.015 | Susceptible | 0.5 | Wildtype |
| MYC-19-0192 | CAD64 | SAMN51609427 | West | 2018 | Abdomen | 8 | Susceptible dose | 0.015 | Susceptible | 1 | Wildtype |
| MYC-19-0193 | CAD65 | SAMN51609428 | West | 2018 | Blood | 8 | Susceptible dose | ≤ 0.008 | Susceptible | 0.5 | Wildtype |
| MYC-19-0194 | CAD66 | SAMN51609429 | West | 2018 | Blood | 8 | Susceptible dose | ≤ 0.008 | Susceptible | 1 | Wildtype |
| MYC-19-0195 | CAD67 | SAMN51609430 | West | 2018 | Blood | 4 | Susceptible dose | 0.015 | Susceptible | 2 | Wildtype |
| MYC-19-0207 | CAD68 | SAMN51609431 | West | 2019 | Blood | 8 | Susceptible dose | 0.015 | Susceptible | 0.5 | Wildtype |
| MYC-19-0208 | CAD69 | SAMN51609432 | West | 2019 | Blood - peripheral | 8 | Susceptible dose | ≤ 0.008 | Susceptible | 0.5 | Wildtype |
| MYC-19-0209 | CAD70 | SAMN51609433 | West | 2019 | Blood | 8 | Susceptible dose | 0.015 | Susceptible | 0.5 | Wildtype |
| MYC-19-0210 | CAD71 | SAMN51609434 | West | 2019 | Blood | 4 | Susceptible dose | 0.015 | Susceptible | 0.5 | Wildtype |
| MYC-19-0211 | CAD72 | SAMN51609435 | West | 2019 | Blood | 4 | Susceptible dose | 0.015 | Susceptible | 0.25 | Wildtype |
| MYC-19-0213 | CAD73 | SAMN51609436 | West | 2019 | Tissue - liver | 4 | Susceptible dose | 0.008 | Susceptible | 0.5 | Wildtype |
| MYC-19-0215 | CAD74 | SAMN51609437 | West | 2019 | Isolate - hip, left | 8 | Susceptible dose | 0.015 | Susceptible | 0.25 | Wildtype |
| MYC-19-0217 | CAD75 | SAMN51609438 | West | 2019 | Peritoneal fluid | 8 | Susceptible dose | 0.015 | Susceptible | 0.5 | Wildtype |
| MYC-19-0220 | CAD76 | SAMN51609439 | Central West | 2019 | Blood | 8 | Susceptible dose | 0.015 | Susceptible | 1 | Wildtype |
| MYC-19-0221 | CAD77 | SAMN51609440 | West | 2019 | Peritoneal fluid | 8 | Susceptible dose | 0.015 | Susceptible | 0.5 | Wildtype |
| MYC-19-0227 | CAD78 | SAMN51609441 | West | 2019 | Kidney | 8 | Susceptible dose | ≤ 0.008 | Susceptible | 0.25 | Wildtype |
| MYC-19-0229 | CAD79 | SAMN51609442 | Central West | 2019 | Blood | 8 | Susceptible dose | 0.015 | Susceptible | 0.5 | Wildtype |
| MYC-19-0231 | CAD80 | SAMN51609443 | East | 2019 | Blood | 16 | Susceptible dose | 0.015 | Susceptible | 0.25 | Wildtype |
| MYC-19-0232 | CAD81 | SAMN51609444 | East | 2019 | Blood | 8 | Susceptible dose | 0.008 | Susceptible | 0.5 | Wildtype |
| MYC-19-0233 | CAD82 | SAMN51609445 | East | 2019 | Blood | 8 | Susceptible dose | ≤ 0.008 | Susceptible | 1 | Wildtype |
| MYC-19-0234 | CAD83 | SAMN51609446 | East | 2019 | Blood | 4 | Susceptible dose | ≤ 0.008 | Susceptible | 1 | Wildtype |
| MYC-19-0235 | CAD84 | SAMN51609447 | East | 2019 | Blood | 4 | Susceptible dose | ≤ 0.008 | Susceptible | 0.5 | Wildtype |
| MYC-19-0236 | CAD85 | SAMN51609448 | East | 2019 | Blood | 8 | Susceptible dose | 0.008 | Susceptible | 0.5 | Wildtype |
| MYC-19-0237 | CAD86 | SAMN51609449 | East | 2019 | Blood | 4 | Susceptible dose | 0.008 | Susceptible | 0.25 | Wildtype |
| MYC-19-0238 | CAD87 | SAMN51609450 | East | 2019 | Blood | 64 | Resistant | 0.015 | Susceptible | 0.5 | Wildtype |
| MYC-19-0239 | CAD88 | SAMN51609451 | East | 2019 | Blood | 4 | Susceptible dose | 0.008 | Susceptible | 0.5 | Wildtype |
| MYC-19-0240 | CAD89 | SAMN51609452 | East | 2019 | Blood | 8 | Susceptible dose | 0.015 | Susceptible | 1 | Wildtype |
| MYC-19-0241 | CAD90 | SAMN51609453 | East | 2018 | Blood | 32 | Susceptible dose | < 0.06 | Susceptible | 0.25 | Wildtype |
| MYC-19-0266 | CAD91 | SAMN51609454 | Central West | 2015 | Abdominal abscess | 16 | Susceptible dose | 0.12 | Intermediate | 0.5 | Wildtype |
| MYC-19-0267 | CAD92 | SAMN51609455 | Central West | 2018 | Lower left quadrant aspirate | 8 | Susceptible dose | 0.03 | Susceptible | 1 | Wildtype |
| MYC-19-0268 | CAD93 | SAMN51609456 | Central West | 2018 | Blood culture | 16 | Susceptible dose | 0.06 | Susceptible | 2 | Wildtype |
| MYC-19-0269 | CAD94 | SAMN51609457 | Central West | 2018 | Blood culture | 4 | Susceptible dose | 0.5 | Resistant | 1 | Wildtype |
| MYC-19-0270 | CAD95 | SAMN51609458 | Central West | 2018 | Blood culture | 4 | Susceptible dose | 0.06 | Susceptible | 2 | Wildtype |
| MYC-19-0271 | CAD96 | SAMN51609459 | Central West | 2017 | Wound | 8 | Susceptible dose | 1 | Resistant | 1 | Wildtype |
| MYC-19-0272 | CAD97 | SAMN51609460 | Central West | 2017 | Blood | 4 | Susceptible dose | 0.12 | Intermediate | 4 | Non-wildtype |
| MYC-19-0273 | CAD98 | SAMN51609461 | Central West | 2017 | Blood | 128 | Resistant | 0.03 | Susceptible | 1 | Wildtype |
| MYC-19-0274 | CAD99 | SAMN51609462 | Central West | 2017 | Blood | 64 | Resistant | 0.06 | Susceptible | 2 | Wildtype |
| MYC-19-0275 | CAD100 | SAMN51609463 | Central West | 2019 | Nephrostomy, left | 128 | Resistant | 0.03 | Susceptible | 1 | Wildtype |
| MYC-19-0289 | CAD101 | SAMN51609464 | East | 2018 | Blood | 4 | Susceptible dose | 0.008 | Susceptible | 0.5 | Wildtype |
| MYC-19-0291 | CAD102 | SAMN51609465 | East | 2018 | Blood | 16 | Susceptible dose | 0.008 | Susceptible | 1 | Wildtype |
| MYC-19-0292 | CAD103 | SAMN51609466 | East | 2017 | Blood | 2 | Susceptible dose | 0.008 | Susceptible | 1 | Wildtype |
| MYC-19-0297 | CAD104 | SAMN51609467 | East | 2019 | Blood | 128 | Resistant | 0.008 | Susceptible | 1 | Wildtype |
| MYC-19-0298 | CAD105 | SAMN51609468 | East | 2019 | Blood | 8 | Susceptible dose | ≤ 0.008 | Susceptible | 1 | Wildtype |
| MYC-19-0303 | CAD106 | SAMN51609469 | East | 2014 | Blood | 4 | Susceptible dose | ≤ 0.06 | Susceptible | ≤ 0.25 | Wildtype |
| MYC-19-0304 | CAD107 | SAMN51609470 | East | 2018 | Blood | 4 | Susceptible dose | 0.008 | Susceptible | 1 | Wildtype |
| MYC-19-0306 | CAD108 | SAMN51609471 | East | 2018 | Blood | 32 | Susceptible dose | 0.015 | Susceptible | 1 | Wildtype |
| MYC-19-0308 | CAD109 | SAMN51609472 | East | 2018 | Blood | 4 | Susceptible dose | 0.008 | Susceptible | 0.5 | Wildtype |
| MYC-19-0315 | CAD110 | SAMN51609473 | Central East | 2018 | Abdomen fluid | 4 | Susceptible dose | 0.12 | Intermediate | 0.25 | Wildtype |
| MYC-19-0316 | CAD111 | SAMN51609474 | Central East | 2019 | Pus | 8 | Susceptible dose | 0.06 | Susceptible | 0.5 | Wildtype |
| MYC-19-0317 | CAD112 | SAMN51609475 | Central East | 2018 | Fluid | 64 | Resistant | 0.008 | Susceptible | 1 | Wildtype |
| MYC-19-0318 | CAD113 | SAMN51609476 | Central East | 2018 | Urogenital pus | 64 | Resistant | 0.015 | Susceptible | 0.5 | Wildtype |
| MYC-19-0319 | CAD114 | SAMN51609477 | Central East | 2018 | Ascitic fluid | 128 | Resistant | 0.03 | Susceptible | 1 | Wildtype |
| MYC-19-0320 | CAD115 | SAMN51609478 | Central East | 2018 | Pus | 128 | Resistant | ≤ 0.008 | Susceptible | 0.5 | Wildtype |
| MYC-19-0321 | CAD116 | SAMN51609479 | Central East | 2018 | Hemoculture | > 128 | Resistant | 0.015 | Susceptible | 0.12 | Wildtype |
| MYC-19-0322 | CAD117 | SAMN51609480 | Central East | 2018 | Pleura biopsy | 128 | Resistant | 0.015 | Susceptible | 0.5 | Wildtype |
| MYC-19-0323 | CAD118 | SAMN51609481 | Central East | 2018 | Urine | 64 | Resistant | 0.015 | Susceptible | 0.25 | Wildtype |
| MYC-19-0324 | CAD119 | SAMN51609482 | Central East | 2018 | Tissue | 64 | Resistant | ≤ 0.008 | Susceptible | 0.25 | Wildtype |
| MYC-19-0325 | CAD120 | SAMN51609483 | Central East | 2018 | Urine | 64 | Resistant | 0.015 | Susceptible | 0.25 | Wildtype |
| MYC-19-0326 | CAD121 | SAMN51609484 | Central East | 2018 | Fluid | 64 | Resistant | ≤ 0.008 | Susceptible | 1 | Wildtype |
| MYC-19-0327 | CAD122 | SAMN51609485 | Central East | 2018 | Blood | 64 | Resistant | 0.015 | Susceptible | 0.25 | Wildtype |
| MYC-19-0328 | CAD123 | SAMN51609486 | Central East | 2018 | Urine | > 128 | Resistant | 0.015 | Susceptible | 0.5 | Wildtype |
| MYC-19-0329 | CAD124 | SAMN51609487 | Central East | 2018 | Blood | > 128 | Resistant | 0.03 | Susceptible | 0.5 | Wildtype |
| MYC-19-0330 | CAD125 | SAMN51609488 | Central East | 2018 | Blood | 64 | Resistant | 0.03 | Susceptible | 0.5 | Wildtype |
| MYC-19-0331 | CAD126 | SAMN51609489 | Central East | 2019 | Hemoculture | 128 | Resistant | 0.06 | Susceptible | 0.5 | Wildtype |
| MYC-19-0332 | CAD127 | SAMN51609490 | Central East | 2019 | Bile | 128 | Resistant | 0.015 | Susceptible | 0.5 | Wildtype |
| MYC-19-0333 | CAD128 | SAMN51609491 | Central East | 2019 | Blood | 128 | Resistant | 0.03 | Susceptible | 1 | Wildtype |
| MYC-19-0334 | CAD129 | SAMN51609492 | Central East | 2019 | Pus | 128 | Resistant | 0.015 | Susceptible | 0.12 | Wildtype |
| MYC-19-0335 | CAD130 | SAMN51609493 | Central East | 2019 | Abdomen pus | > 128 | Resistant | 0.015 | Susceptible | 0.25 | Wildtype |
| MYC-19-0336 | CAD131 | SAMN51609494 | Central East | 2019 | Abdomen drain | 128 | Resistant | 0.015 | Susceptible | 0.5 | Wildtype |
| MYC-19-0337 | CAD132 | SAMN51609495 | Central East | 2019 | Hemoculture | 128 | Resistant | 0.015 | Susceptible | 0.12 | Wildtype |
| MYC-19-0338 | CAD133 | SAMN51609496 | Central East | 2019 | Urine | 128 | Resistant | ≤ 0.008 | Susceptible | 0.12 | Wildtype |
| MYC-19-0339 | CAD134 | SAMN51609497 | Central East | 2019 | Blood | 128 | Resistant | 0.015 | Susceptible | 1 | Wildtype |
| MYC-19-0340 | CAD135 | SAMN51609498 | Central East | 2019 | Urine | 64 | Resistant | 0.015 | Susceptible | 1 | Wildtype |
| MYC-19-0341 | CAD136 | SAMN51609499 | Central East | 2019 | Abdomen abscess | > 128 | Resistant | 0.015 | Susceptible | 1 | Wildtype |
| MYC-19-0342 | CAD137 | SAMN51609500 | Central East | 2019 | Gum pus | 64 | Resistant | 0.015 | Susceptible | 0.5 | Wildtype |
| MYC-19-0343 | CAD138 | SAMN51609501 | Central East | 2019 | Pancreatic biopsy - tissue | 128 | Resistant | 0.015 | Susceptible | 0.5 | Wildtype |
| MYC-19-0344 | CAD139 | SAMN51609502 | Central East | 2019 | Urine | 128 | Resistant | 0.03 | Susceptible | 1 | Wildtype |
| MYC-19-0345 | CAD140 | SAMN51609503 | Central East | 2019 | Secretions | 64 | Resistant | ≤ 0.008 | Susceptible | 0.25 | Wildtype |
| MYC-19-0346 | CAD141 | SAMN51609504 | Central East | 2019 | Urine | 64 | Resistant | 0.015 | Susceptible | 1 | Wildtype |
| MYC-19-0386 | CAD142 | SAMN51609505 | Central East | 2020 | Urine | 8 | Susceptible dose | 1 | Resistant | 0.25 | Wildtype |

**Supplementary Table S2. List list of global Candida glabrata isolates (n = 139) included in this study.**

| **BioProject** | **SRR** | **Global ID (this study)** | **Country** | **FLZ (**𝝁**g/mL)** | **FLZ INT** | **MFG (**𝝁**g/mL)** | **MFG INT** | **CAS (**𝝁**g/mL)** | **CAS INT** | **AMB (**𝝁**g/mL)** | **AMB INT** |
| --- | --- | --- | --- | --- | --- | --- | --- | --- | --- | --- | --- |
| PRJNA480138 | SRR8068012 | AUS1 | Australia | 0.5 | Susceptible dose | NA | NA | 0.25 | Intermediate | 1 | Wildtype |
|  | SRR8068013 | AUS2 | Australia | 128 | Resistant | NA | NA | 0.12 | Susceptible | 1 | Wildtype |
|  | SRR8068014 | AUS3 | Australia | 16 | Susceptible dose | NA | NA | 0.06 | Susceptible | 0.5 | Wildtype |
|  | SRR8068015 | AUS4 | Australia | 16 | Susceptible dose | NA | NA | 0.06 | Susceptible | 1 | Wildtype |
|  | SRR8068016 | AUS5 | Australia | 32 | Susceptible dose | NA | NA | 0.06 | Susceptible | 1 | Wildtype |
|  | SRR8068017 | AUS6 | Australia | 16 | Susceptible dose | NA | NA | 0.25 | Intermediate | 1 | Wildtype |
|  | SRR8068018 | AUS7 | Australia | 8 | Susceptible dose | NA | NA | 0.06 | Susceptible | 1 | Wildtype |
|  | SRR8068019 | AUS8 | Australia | 256 | Resistant | NA | NA | 0.12 | Susceptible | 0.5 | Wildtype |
|  | SRR8068020 | AUS9 | Australia | 4 | Susceptible dose | NA | NA | 0.12 | Susceptible | 0.25 | Wildtype |
|  | SRR8068021 | AUS10 | Australia | 128 | Resistant | NA | NA | 0.06 | Susceptible | 1 | Wildtype |
|  | SRR8068022 | AUS11 | Australia | 4 | Susceptible dose | NA | NA | 8 | Resistant | 0.5 | Wildtype |
|  | SRR8068023 | AUS12 | Australia | 16 | Susceptible dose | NA | NA | 0.03 | Susceptible | 0.25 | Wildtype |
|  | SRR8068024 | AUS13 | Australia | 64 | Resistant | NA | NA | 0.06 | Susceptible | 0.25 | Wildtype |
|  | SRR8068025 | AUS14 | Australia | 32 | Susceptible dose | NA | NA | 1 | Resistant | 0.25 | Wildtype |
|  | SRR8068026 | AUS15 | Australia | 16 | Susceptible dose | NA | NA | 0.25 | Intermediate | 1 | Wildtype |
|  | SRR8068028 | AUS16 | Australia | 256 | Resistant | NA | NA | 0.12 | Susceptible | 1 | Wildtype |
|  | SRR8068029 | AUS17 | Australia | 8 | Susceptible dose | NA | NA | >8 | Resistant | 2 | Wildtype |
|  | SRR8068030 | AUS18 | Australia | 128 | Resistant | NA | NA | 0.06 | Susceptible | 0.5 | Wildtype |
|  | SRR8068031 | AUS19 | Australia | 1 | Susceptible dose | NA | NA | 0.03 | Susceptible | 0.5 | Wildtype |
|  | SRR8068032 | AUS20 | Australia | 16 | Susceptible dose | NA | NA | 0.5 | Resistant | 1 | Wildtype |
|  | SRR8068033 | AUS21 | Australia | 32 | Susceptible dose | NA | NA | 0.25 | Intermediate | 0.25 | Wildtype |
|  | SRR8068034 | AUS22 | Australia | 32 | Susceptible dose | NA | NA | 0.06 | Susceptible | 0.25 | Wildtype |
|  | SRR8068035 | AUS23 | Australia | 128 | Resistant | NA | NA | 0.008 | Susceptible | 1 | Wildtype |
|  | SRR8068036 | AUS24 | Australia | 8 | Susceptible dose | NA | NA | 0.008 | Susceptible | 0.125 | Wildtype |
|  | SRR8068037 | AUS25 | Australia | 32 | Susceptible dose | NA | NA | 0.25 | Intermediate | 0.06 | Wildtype |
|  | SRR8068038 | AUS26 | Australia | 64 | Resistant | NA | NA | 0.25 | Intermediate | 0.25 | Wildtype |
|  | SRR8068039 | AUS27 | Australia | 16 | Susceptible dose | NA | NA | 0.12 | Susceptible | 1 | Wildtype |
|  | SRR8068040 | AUS28 | Australia | 32 | Susceptible dose | NA | NA | 0.25 | Intermediate | 0.06 | Wildtype |
|  | SRR8068041 | AUS29 | Australia | 4 | Susceptible dose | NA | NA | 0.25 | Intermediate | 1 | Wildtype |
|  | SRR8068042 | AUS30 | Australia | 128 | Resistant | NA | NA | 0.125 | Susceptible | 0.125 | Wildtype |
|  | SRR8068043 | AUS31 | Australia | 256 | Resistant | NA | NA | 0.25 | Intermediate | 0.25 | Wildtype |
|  | SRR8068044 | AUS32 | Australia | 8 | Susceptible dose | NA | NA | 0.06 | Susceptible | 1 | Wildtype |
|  | SRR8068045 | AUS33 | Australia | 8 | Susceptible dose | NA | NA | 0.12 | Susceptible | 1 | Wildtype |
|  | SRR8068046 | AUS34 | Australia | 16 | Susceptible dose | NA | NA | 0.12 | Susceptible | 0.5 | Wildtype |
|  | SRR8068047 | AUS35 | Australia | 16 | Susceptible dose | NA | NA | 0.06 | Susceptible | 1 | Wildtype |
|  | SRR8068048 | AUS36 | Australia | 4 | Susceptible dose | NA | NA | 0.03 | Susceptible | 0.5 | Wildtype |
|  | SRR8068049 | AUS37 | Australia | 128 | Resistant | NA | NA | 0.06 | Susceptible | 1 | Wildtype |
|  | SRR8068050 | AUS38 | Australia | 8 | Susceptible dose | NA | NA | 0.12 | Susceptible | 0.5 | Wildtype |
|  | SRR8068051 | AUS39 | Australia | 8 | Susceptible dose | NA | NA | 0.25 | Intermediate | 1 | Wildtype |
|  | SRR8068052 | AUS40 | Australia | 2 | Susceptible dose | NA | NA | 0.06 | Susceptible | 0.5 | Wildtype |
|  | SRR8068053 | AUS41 | Australia | 64 | Resistant | NA | NA | 0.12 | Susceptible | 0.5 | Wildtype |
|  | SRR8068054 | AUS42 | Australia | 256 | Resistant | NA | NA | 0.25 | Intermediate | 0.125 | Wildtype |
|  | SRR8068055 | AUS43 | Australia | 8 | Susceptible dose | NA | NA | 0.25 | Intermediate | 1 | Wildtype |
|  | SRR8068056 | AUS44 | Australia | 32 | Susceptible dose | NA | NA | 0.12 | Susceptible | 0.5 | Wildtype |
|  | SRR8068057 | AUS45 | Australia | 8 | Susceptible dose | NA | NA | 0.12 | Susceptible | 0.5 | Wildtype |
|  | SRR8068058 | AUS46 | Australia | 16 | Susceptible dose | NA | NA | 0.12 | Susceptible | 0.5 | Wildtype |
|  | SRR8068059 | AUS47 | Australia | 8 | Susceptible dose | NA | NA | 0.12 | Susceptible | 0.5 | Wildtype |
|  | SRR8068060 | AUS48 | Australia | 32 | Susceptible dose | NA | NA | 0.06 | Susceptible | 1 | Wildtype |
|  | SRR8068061 | AUS49 | Australia | 2 | Susceptible dose | NA | NA | 2 | Resistant | 0.5 | Wildtype |
|  | SRR8068062 | AUS50 | Australia | 4 | Susceptible dose | NA | NA | 2 | Resistant | 0.5 | Wildtype |
| PRJNA361477 | SRR5239758 | FRA1 | France | 8 | Susceptible dose | 0.032-0.250 | Susceptible | NA | NA | 0.004 | Wildtype |
|  | SRR5239756 | BEL1 | Belgium | 8 | Susceptible dose | 0.032-0.064 | Susceptible | NA | NA | 0.004 | Wildtype |
|  | SRR5239759 | GER1 | Germany | 16 | Susceptible dose | 0.032-0.064 | Susceptible | NA | NA | 0.004 | Wildtype |
|  | SRR5239760 | USA1 | USA | 4-8 | Susceptible dose | 0.032-0.250 | Susceptible | NA | NA | 0.004 | Wildtype |
|  | SRR5239761 | USA2 | USA | 128 | Resistant | 0.032-0.064 | Susceptible | NA | NA | 0.004 | Wildtype |
|  | SRR5239754 | FRA2 | France | 8 | Susceptible dose | 0.032-0.064 | Susceptible | NA | NA | 0.004 | Wildtype |
|  | SRR5239755 | FRA3 | France | 8 | Susceptible dose | 0.032-0.064 | Susceptible | NA | NA | 0.004 | Wildtype |
|  | SRR5239757 | USA3 | USA | 16 | Susceptible dose | 0.032-0.064 | Susceptible | NA | NA | 0.004-0.008 | Wildtype |
|  | SRR5239762 | TWN1 | Taiwan | 8-16 | Susceptible dose | 0.032-0.064 | Susceptible | NA | NA | 0.004 | Wildtype |
|  | SRR5239763 | TWN2 | Taiwan | 4-16 | Susceptible dose | 0.032-0.064 | Susceptible | NA | NA | 0.004 | Wildtype |
|  | SRR5239764 | FRA4 | France | 4 | Susceptible dose | 0.032-0.064 | Susceptible | NA | NA | 0.004 | Wildtype |
|  | SRR5239765 | FRA5 | France | 4-8 | Susceptible dose | 0.032-0.250 | Susceptible | NA | NA | 0.004 | Wildtype |
|  | SRR5239766 | FRA6 | France | 4-8 | Susceptible dose | 0.032-0.064 | Susceptible | NA | NA | 0.004 | Wildtype |
|  | SRR5239767 | FRA7 | France | 8 | Susceptible dose | 0.032-0.064 | Susceptible | NA | NA | 0.004-0.008 | Wildtype |
|  | SRR5239768 | FRA8 | France | 4-8 | Susceptible dose | 0.032-0.064 | Susceptible | NA | NA | 0.004 | Wildtype |
|  | SRR5239769 | ITA1 | Italy | 2-4 | Susceptible dose | 0.032-0.064 | Susceptible | NA | NA | 0.004 | Wildtype |
|  | SRR5239770 | ITA2 | Italy | 1-4 | Susceptible dose | 0.032-0.064 | Susceptible | NA | NA | 0.004 | Wildtype |
|  | SRR5239771 | USA4 | USA | 4-8 | Susceptible dose | 0.032-0.064 | Susceptible | NA | NA | 0.004 | Wildtype |
|  | SRR5239772 | USA5 | USA | 8 | Susceptible dose | 0.032-0.064 | Susceptible | NA | NA | 0.004 | Wildtype |
|  | SRR5239773 | FRA9 | France | 4-8 | Susceptible dose | 0.032-0.064 | Susceptible | NA | NA | 0.004 | Wildtype |
|  | SRR5239753 | USA6 | USA | 8 | Susceptible dose | 0.032-0.064 | Susceptible | NA | NA | 0.004 | Wildtype |
|  | SRR5239774 | USA7 | USA | 8 | Susceptible dose | 0.032-0.064 | Susceptible | NA | NA | 0.004 | Wildtype |
|  | SRR5239775 | USA8 | USA | 8-16 | Susceptible dose | 0.032 | Susceptible | NA | NA | 0.004-0.008 | Wildtype |
|  | SRR5239776 | BEL2 | Belgium | 8 | Susceptible dose | 0.032-0.125 | Susceptible | NA | NA | 0.004 | Wildtype |
|  | SRR5239777 | USA9 | USA | 4 | Susceptible dose | 0.032-0.064 | Susceptible | NA | NA | 0.004-0.032 | Wildtype |
|  | SRR5239778 | USA10 | USA | 16 | Susceptible dose | 0.032-0.064 | Susceptible | NA | NA | 0.004 | Wildtype |
|  | SRR5239779 | USA11 | USA | 64 | Resistant | 0.032-0.250 | Susceptible | NA | NA | 0.004 | Wildtype |
|  | SRR5239780 | USA12 | USA | 8 | Susceptible dose | 0.032-0.064 | Susceptible | NA | NA | 0.004 | Wildtype |
|  | SRR5239781 | BEL3 | Belgium | 4-8 | Susceptible dose | 0.032-0.125 | Susceptible | NA | NA | 0.004 | Wildtype |
|  | SRR5239782 | BEL4 | Belgium | 8 | Susceptible dose | 0.032-0.064 | Susceptible | NA | NA | 0.004-0.008 | Wildtype |
|  | SRR5239783 | BEL5 | Belgium | 4 | Susceptible dose | 0.032-0.064 | Susceptible | NA | NA | 0.004 | Wildtype |
|  | SRR5239784 | BEL6 | Belgium | 2-4 | Susceptible dose | 0.032-0.125 | Susceptible | NA | NA | 0.004 | Wildtype |
| PRJEB40738 | ERR4669795 | DNK1 | Denmark | 16 | Susceptible dose | 0.015 | Susceptible | NA | NA | 1 | Wildtype |
|  | ERR4669789 | DNK2 | Denmark | 128 | Resistant | 0.015 | Susceptible | NA | NA | 1 | Wildtype |
|  | ERR4669787 | DNK3 | Denmark | 4 | Susceptible dose | ≤0.008 | Susceptible | NA | NA | 0.5 | Wildtype |
|  | ERR4669786 | DNK4 | Denmark | 16 | Susceptible dose | 0.015 | Susceptible | NA | NA | 0.5 | Wildtype |
|  | ERR4669784 | DNK5 | Denmark | 128 | Resistant | 0.015 | Susceptible | NA | NA | 0.5 | Wildtype |
|  | ERR4669782 | DNK6 | Denmark | 6 | Susceptible dose | 0.015 | Susceptible | NA | NA | 1 | Wildtype |
|  | ERR4669779 | DNK7 | Denmark | 4 | Susceptible dose | ≤0.008 | Susceptible | NA | NA | 1 | Wildtype |
|  | ERR4669757 | DNK8 | Denmark | 1 | Susceptible dose | ≤0.008 | Susceptible | NA | NA | 0.5 | Wildtype |
| PRJNA592373 | SRR25168788 | USA13 | USA | 64 | Resistant | 1 | Resistant | NA | NA | 0.5 | Wildtype |
|  | SRR25168783 | USA15 | USA | 4 | Susceptible dose | 0.25 | Resistant | NA | NA | 0.06 | Wildtype |
|  | SRR25168787 | USA16 | USA | 32 | Susceptible dose | 4 | Resistant | NA | NA | 0.25 | Wildtype |
|  | SRR25168785 | USA17 | USA | 4 | Susceptible dose | 2 | Resistant | NA | NA | 0.25 | Wildtype |
|  | SRR25168791 | USA18 | USA | 4 | Susceptible dose | 0.25 | Resistant | NA | NA | 0.25 | Wildtype |
|  | SRR25168784 | USA20 | USA | 8 | Susceptible dose | 0.25 | Resistant | NA | NA | 0.25 | Wildtype |
|  | SRR25168792 | USA21 | USA | 4 | Susceptible dose | 4 | Resistant | NA | NA | 0.25 | Wildtype |
|  | SRR25168789 | USA22 | USA | 8 | Susceptible dose | 2 | Resistant | NA | NA | 0.25 | Wildtype |
|  | SRR25168786 | USA23 | USA | 128 | Resistant | 4 | Resistant | NA | NA | 0.5 | Wildtype |
|  | SRR25168790 | USA24 | USA | 4 | Susceptible dose | 0.015 | Susceptible | NA | NA | 0.03 | Wildtype |
|  | SRR25177397 | USA25 | USA | 16 | Susceptible dose | 0.015 | Susceptible | NA | NA | 0.25 | Wildtype |
|  | SRR25177394 | USA26 | USA | 8 | Susceptible dose | 0.015 | Susceptible | NA | NA | 0.25 | Wildtype |
|  | SRR25177398 | USA27 | USA | 8 | Susceptible dose | 0.03 | Susceptible | NA | NA | 0.25 | Wildtype |
|  | SRR25177402 | USA28 | USA | 8 | Susceptible dose | 0.015 | Susceptible | NA | NA | 0.25 | Wildtype |
|  | SRR25177401 | USA30 | USA | 64 | Resistant | 0.03 | Susceptible | NA | NA | 0.25 | Wildtype |
|  | SRR25169060 | USA32 | USA | 128 | Resistant | 0.008 | Susceptible | NA | NA | 0.5 | Wildtype |
|  | SRR25169059 | USA33 | USA | 64 | Resistant | 1 | Resistant | NA | NA | 0.25 | Wildtype |
|  | SRR25169057 | USA34 | USA | 8 | Susceptible dose | 0.016 | Susceptible | NA | NA | 0.5 | Wildtype |
|  | SRR25169056 | USA35 | USA | 8 | Susceptible dose | 0.016 | Susceptible | NA | NA | 0.25 | Wildtype |
|  | SRR25169058 | USA36 | USA | 8 | Susceptible dose | 4 | Resistant | NA | NA | 0.12 | Wildtype |
| PRJNA310957 | SRR3154238 | AUS51 | Australia | 8 | Susceptible dose | <0.008 | Susceptible | NA | NA | 0.5 | Wildtype |
|  | SRR3154237 | AUS52 | Australia | 4 | Susceptible dose | 1 | Resistant | NA | NA | 0.5 | Wildtype |
|  | SRR3154236 | AUS53 | Australia | 8 | Susceptible dose | 0.015 | Susceptible | NA | NA | 0.25 | Wildtype |
|  | SRR3154235 | AUS54 | Australia | 8 | Susceptible dose | 1 | Resistant | NA | NA | 2 | Wildtype |
|  | SRR3151582 | AUS55 | Australia | 256 | Resistant | 0.015 | Susceptible | NA | NA | 1 | Wildtype |
|  | SRR4237306 | AUS56 | Australia | 256 | Resistant | 0.015 | Susceptible | NA | NA | 0.25 | Wildtype |
|  | SRR4198629 | AUS57 | Australia | 4 | Susceptible dose | 0.008 | Susceptible | NA | NA | 0.5 | Wildtype |
|  | SRR3151534 | AUS58 | Australia | 16 | Susceptible dose | 0.015 | Susceptible | NA | NA | 1 | Wildtype |
|  | SRR3151533 | AUS59 | Australia | 16 | Susceptible dose | <0.008 | Susceptible | NA | NA | 1 | Wildtype |
|  | SRR3154234 | AUS60 | Australia | 8 | Susceptible dose | <0.008 | Susceptible | NA | NA | 0.5 | Wildtype |
|  | SRR3154166 | AUS61 | Australia | 128 | Resistant | 0.015 | Susceptible | NA | NA | 0.5 | Wildtype |
|  | SRR3146184 | AUS62 | Australia | 8 | Susceptible dose | 0.015 | Susceptible | NA | NA | 1 | Wildtype |
| PRJNA610214 | SRR11235421 | CAD143 | Canada | NA | NA | 0.12 | Intermediate | 0.5 | Resistant | NA | NA |
|  | SRR11235422 | CAD144 | Canada | NA | NA | 0.015 | Susceptible | 0.06 | Susceptible | NA | NA |
|  | SRR11235423 | CAD145 | Canada | NA | NA | 0.12 | Intermediate | 0.25 | Intermediate | NA | NA |
|  | SRR11235424 | CAD146 | Canada | NA | NA | 0.015 | Susceptible | 0.12 | Susceptible | NA | NA |
|  | SRR11235425 | CAD147 | Canada | NA | NA | 8 | Resistant | 8 | Resistant | NA | NA |
|  | SRR11235426 | CAD148 | Canada | NA | NA | 0.015 | Susceptible | 0.06 | Susceptible | NA | NA |
|  | SRR11235427 | CAD149 | Canada | NA | NA | 2 | Resistant | 8 | Resistant | NA | NA |
|  | SRR11235428 | CAD150 | Canada | NA | NA | 0.03 | Susceptible | 0.12 | Susceptible | NA | NA |
|  | SRR11235429 | CAD151 | Canada | NA | NA | NA | NA | 0.5 | Resistant | NA | NA |
| PRJNA483064 | SRR7609359 | GER2 | Germany | >64 | Resistant | >8 | Resistant | NA | NA | 0.5 | Wildtype |
|  | SRR7609360 | GER3 | Germany | >64 | Resistant | 0.5 | Resistant | NA | NA | 0.25 | Wildtype |
| PRJNA374542 | SRR5459193 | SWI1 | Switzerland | 4 | Susceptible dose | NA | NA | NA | NA | 0.5 | Wildtype |
|  | SRR5459192 | SWI2 | Switzerland | 64 | Resistant | NA | NA | NA | NA | 0.5 | Wildtype |
| PRJNA524686 | SRR8697501 | USA31 | USA | 128 | Resistant | 0.03 | Susceptible | NA | NA | 0.12 | Wildtype |
|  | SRR8697375 | USA14 | USA | 4 | Susceptible dose | 4 | Resistant | NA | NA | 0.5 | Wildtype |
|  | SRR8697341 | USA29 | USA | 64 | Resistant | 0.015 | Susceptible | NA | NA | 0.25 | Wildtype |
| PRJNA329124 | SRR3925758 | USA19 | USA | 64 | Resistant | 1 | Resistant | NA | NA | 0.12 | Wildtype |

**Supplementary Table S3. Within and between clade single nucleotide variant (SNV) diversity among fifteen clusters of clinical *Candida glabrata* isolates (n = 142) from Canada, showing minimum, median, and maximum pairwise SNV differences.**

|  | Intra-clade SNV diversity | | | Inter-clade SNV diversity | | |
| --- | --- | --- | --- | --- | --- | --- |
| Cluster (n) | Minimum | Median | Maximum | Minimum | Median | Maximum |
| I (29) | 2 | 388 | 526 | 20,345 | 24,493 | 27,300 |
| II (3) | 179 | 180 | 211 | 20,041 | 24,242 | 27,391 |
| III (22) | 3 | 336 | 514 | 19,784 | 23,537 | 27,199 |
| IV (4) | 431 | 603 | 783 | 15,140 | 23,088 | 26,590 |
| V (3) | 399 | 515 | 672 | 16,747 | 24,163 | 25,164 |
| VI (4) | 40 | 223 | 399 | 15,955 | 23,956 | 24,979 |
| VII (6) | 315 | 418 | 609 | 15,678 | 24,151 | 25,281 |
| VIII (12) | 2 | 406 | 681 | 13,564 | 24,076 | 24,872 |
| IX (6) | 181 | 246 | 554 | 20,662 | 24,395 | 26,947 |
| X (15) | 13 | 546 | 936 | 20,484 | 24,599 | 27,279 |
| XI (2) | 297 | 297 | 297 | 6910 | 24,279 | 26,768 |
| XII (2) | 200 | 200 | 200 | 6910 | 24,264 | 27,058 |
| XIII (3) | 2 | 35 | 37 | 14,837 | 24,135 | 26,742 |
| XIV (4) | 300 | 378 | 428 | 17,345 | 24,144 | 27,019 |
| XV (15) | 59 | 372 | 634 | 16,066 | 24,250 | 26,925 |

SNV, single nucleotide variant.

**Supplementary Table S4. Variants identified in known genes associated with antifungal resistance in the Canadian *Candida glabrata* collection.**

| **Isolate ID** | ***PDR1* (CAGL0A00451g)** | ***CDR1* (CAGL0M01760g)** | ***PDH1* (*CDR2*) (CAGL0F02717g)** | ***SNQ2* (CAGL0I04862g)** | ***UPC2A* (CAGL0C01199g)** | ***ERG2* (CAGL0L10714g)** | ***ERG3* (CAGL0F01793g)** | ***ERG6* (CAGL0H04653g)** | ***ERG11* (CAGL0E04334g)** | ***FKS1* (CAGL0G01034g)** | ***FKS2* (CAGL0K04037g)** | ***FKS3* ( CAGL0M13827g)** | ***SUR4* (CAGL0G04851g)** | ***SUR2* (CAGL0H01375g)** | ***IFA38* (CAGL0H07513g)** | ***FEN1* (CAGL0L08184g)** | ***MSH2* (CAGL0I07733g)** |
| --- | --- | --- | --- | --- | --- | --- | --- | --- | --- | --- | --- | --- | --- | --- | --- | --- | --- |
| MYC-19-0003 | V91I, L98S, V134A | T16I, H58Y, H271R | E839D | K1156Q, H1147Q | M922V, N304S, A254V, R92K | I207V | I207V | WT | WT | WT | WT | T1676S | WT | WT | WT | WT | E478Q |
| MYC-19-0004 | S76P, V91I, L98S, T143P | WT | E839D | WT | N323Y, N304S | I207V | I207V | WT | WT | WT | WT | I3T, R1472Q, I1512V, A1621T, Y1731C, I1751V, S1840Y | WT | WT | WT | WT | V239L, A942T |
| MYC-19-0007 | S76P, V91I, L98S, T143P | WT | E839D, E863D | WT | E540Q, N304S, T260S, A167G, R92K | I207V | I207V | R48K | WT | WT | Ter1898Ter | I3T, R1472Q, I1512V, Y1731C, I1751V, R1761M, S1840Y | WT | WT | I189V | WT | WT |
| MYC-19-0008 | S76P, V91I, L98S, T143P | WT | E839D, E863D | WT | E540Q, N304S, T260S, A167G, R92K | I207V | I207V | WT | WT | WT | Ter1898Ter | I3T, R1472Q, I1512V, Y1731C, I1751V, R1761M, S1840Y | WT | WT | I189V | WT | WT |
| MYC-19-0014 | S76P, V91I, L98S, T143P | WT | E839D | WT | WT | I207V | I207V | WT | WT | WT | WT | R1039L, N1825S | WT | WT | I189V | WT | P208S, N890I |
| MYC-19-0015 | S76P, V91I, L98S, T143P, N764I | H1098Y | K438Q, V734I, E839D | G1421S, T613S | N304S, R92K | I207V | I207V | WT | WT | WT | WT | I389V | H158R | WT | WT | V62I | E231G, L269F |
| MYC-19-0016 | S76P, V91I, L98S, T143P | WT | E839D | WT | WT | I207V | I207V | WT | WT | WT | WT | R1039L, N1825S | WT | WT | I189V | WT | P208S, N890I |
| MYC-19-0017 | V91I, L98S, V134A | T16I, H58Y, H271R | E839D | K1156Q, H1147Q | N304S, R92K | I207V | I207V | WT | WT | WT | WT | T1676S | WT | WT | WT | WT | E478Q |
| MYC-19-0018 | S76P, V91I, L98S, T143P | WT | K438Q, V734I, E839D | G1421S, M872V | N304S, R92K | I207V | I207V | WT | WT | WT | WT | I389V | H158R | WT | WT | V62I | E231G, L269F |
| MYC-19-0024 | S76P, V91I, L98S, T143P | WT | E839D | WT | N304S | I207V | I207V | WT | WT | WT | S144F | I3T, R1472Q, I1512V, A1621T, Y1731C, I1751V, S1840Y | WT | WT | WT | WT | V239L, A942T |
| MYC-19-0025 | S76P, V91I, L98S, T143P, R761G | V914I | E839D | WT | E540Q, N304S, T260S, A167G, R92K | I207V | I207V | R48K | WT | WT | WT | I279F, L884V, I1355M, I1469V, R1472Q, I1512V, Y1731C, I1751V | WT | WT | I339M | WT | WT |
| MYC-19-0026 | S76P, V91I, L98S, T143P | H58Y, S1261C | E839D | WT | WT | L60F, I207V | L60F, I207V | WT | WT | WT | E78D, T926P, Ter1898Ter | I3T, R1472Q, I1512V, Y1731C, I1751V, S1840Y | WT | WT | WT | WT | WT |
| MYC-19-0027 | S76P, V91I, L98S, T143P | V914I | E839D | WT | E540Q, N304S, T260S, A167G, R92K | I207V | I207V | R48K | WT | WT | WT | I279F, L884V, I1355M, I1469V, R1472Q, I1512V, Y1731C, I1751V | WT | WT | I339M | WT | WT |
| MYC-19-0044 | S76P, V91I, L98S, T143P | WT | K438Q, V734I, E839D | G1421S | N304S, R92K | I207V | I207V | WT | WT | WT | WT | I389V | H158R | WT | WT | V62I | E231G, L269F |
| MYC-19-0045 | S76P, V91I, L98S, T143P, S391L | V35E, F551L, L554F | F21fs, K438Q, V734I, E839D | G1421S | N304S, R92K | I207V | I207V | WT | WT | L523F | WT | I389V | H158R | WT | WT | V62I | E231G, L269F |
| MYC-19-0046 | S76P, V91I, L98S, T143P | V914I | E839D | WT | E540Q, N304S, T260S, A167G, R92K | I207V | I207V | R48K | WT | WT | WT | I279F, L884V, I1355M, I1469V, R1472Q, I1512V, Y1731C, I1751V | WT | WT | I339M | WT | WT |
| MYC-19-0047 | S76P, V91I, L98S, T143P | V914I | E839D | WT | E540Q, N304S, T260S, A167G, R92K | I207V | I207V | R48K | WT | WT | WT | I279F, L884V, I1355M, I1469V, R1472Q, I1512V, Y1731C, I1751V | WT | WT | I339M | WT | WT |
| MYC-19-0048 | S76P, V91I, L98S, T143P | WT | K438Q, V734I, E839D | G1421S | N304S, R92K | I207V | I207V | WT | WT | WT | WT | I389V | H158R | WT | WT | V62I | L269F |
| MYC-19-0050 | S76P, V91I, L98S, T143P | WT | K438Q, V734I, E839D | G1421S | N304S, R92K | I207V | I207V | WT | WT | WT | WT | I389V | H158R | WT | WT | V62I | E231G, L269F |
| MYC-19-0051 | V91I, L98S, D243N | WT | K438Q, E839D | WT | E822V, P687L, N304S, Q261del, R92K | I207V | I207V | K53N | WT | G14S | T926P | A42V, T1676S | WT | WT | S284F | WT | WT |
| MYC-19-0052 | S76P, V91I, L98S, T143P | H58Y, D445N, S815T, P1105S | E839D | F773S | WT | I207V | I207V | WT | WT | WT | WT | WT | I103V | WT | WT | WT | WT |
| MYC-19-0053 | S76P, V91I, L98S, T143P | H58Y | E839D | WT | WT | L60F, I207V | L60F, I207V | WT | WT | WT | E78D, T926P, Ter1898Ter | I3T, R1472Q, I1512V, Y1731C, I1751V, S1840Y | WT | WT | WT | WT | WT |
| MYC-19-0082 | S76P, V91I, L98S, T143P, R772K, I971V | H58Y | E839D, H1129R | H1147Q | E540Q, N304S, T260S, A167G, R92K | I207V | I207V | WT | L97F | WT | WT | S434fs, L1537M, I1779F | WT | WT | WT | WT | WT |
| MYC-19-0083 | S76P, V91I, L98S, T143P, N764I | H1098Y | K438Q, V734I, E839D | G1421S, T613S | N304S, R92K | I207V | I207V | WT | K152R | WT | WT | I389V | H158R | WT | WT | V62I | E231G, L269F |
| MYC-19-0084 | S76P, V91I, L98S, T143P, T745A | H58Y, N813Y | E839D | WT | E540Q, N304S, T260S, A167G, R92K | I207V | I207V | WT | WT | WT | WT | I3T, A42G, K206E, N865S, R1472Q, F1768I | S362T | WT | I189V | WT | WT |
| MYC-19-0087 | V91I, L98S, D243N, G583S | WT | K438Q, E839D | WT | E822V, P687L, N304S, R92K | I207V | I207V | WT | V155A | G14S | T926P | A42V, T1676S | WT | WT | S284F | WT | WT |
| MYC-19-0088 | S76P, V91I, L98S, T143P, G493D, R592S | H58Y | E839D, E863D | WT | V477I | I207V | I207V | WT | WT | WT | E1149K | V645I, G1009D, R1472Q, I1512V, Y1731C, I1751V, S1840Y | WT | WT | WT | WT | V239L, S757T |
| MYC-19-0089 | S76P, V91I, L98S, T143P, V849L | V914I | E839D, K1130I | WT | E540Q, N304S, T260S, A167G, R92K | I207V | I207V | R48K | WT | WT | WT | I279F, L884V, I1355M, I1469V, R1472Q, I1512V, Y1731C, I1751V | WT | WT | I339M | WT | WT |
| MYC-19-0091 | V91I, L98S, D243N | WT | K438Q, E839D | WT | E822V, N304S, R92K | I207V | I207V | WT | WT | G14S | T926P, K1357T | A42V, T1676S | WT | WT | S284F | WT | WT |
| MYC-19-0092 | S76P, V91I, L98S, T143P, I616F | WT | E839D | WT | WT | I207V | I207V | WT | WT | S629P | Q73* | R1039L, N1825S | WT | WT | I189V | WT | P208S, N890I |
| MYC-19-0093 | V91I, L98S, D243N, K1087E | WT | K438Q, E839D | P1104H | E822V, N304S, R92K | I207V | I207V | WT | WT | G14S | S663P, T926P | A42V, T1676S | WT | WT | S284F | WT | WT |
| MYC-19-0094 | V91I, L98S, D243N | WT | K438Q, E839D | WT | WT | I207V | R208H | WT | WT | G14S | S663P, T926P | A42V, T1676S | WT | WT | S284F | WT | WT |
| MYC-19-0095 | V91I, L98S, D243N | WT | K438Q, E839D | WT | E822V, N304S, R92K | I207V | I207V | WT | WT | G14S | F659del, T926P | A42V, T1676S | WT | WT | S284F | WT | WT |
| MYC-19-0096 | S76P, V91I, L98S, T143P | WT | E839D | WT | N304S | I207V | I207V | WT | WT | WT | WT | I3T, R1472Q, I1512V, A1621T, Y1731C, I1751V, S1840Y | WT | WT | WT | WT | V239L, A942T |
| MYC-19-0097 | S76P, V91I, L98S, T143P | V914I | E839D | WT | E540Q, N304S, T260S, A167G, R92K | I207V | I207V | R48K | WT | WT | F659del | I279F, L884V, I1355M, I1469V, R1472Q, I1512V, Y1731C, I1751V | WT | WT | I339M | WT | WT |
| MYC-19-0098 | S76P, V91I, L98S, T143P | WT | K438Q, V734I, E839D | G1421S | N304S, R92K | I207V | I207V | WT | WT | WT | WT | I389V | H158R | WT | WT | V62I | E231G, L269F |
| MYC-19-0099 | S76P, V91I, L98S, T143P, S391L | A13P, F551L, L554F | K438Q, V734I, E839D | G1421S | N304S, R92K | D83E, I207V | D83E, I207V | WT | WT | WT | WT | I389V | H158R | WT | WT | V62I | E231G, L269F |
| MYC-19-0100 | S76P, V91I, L98S, T143P | H58Y, N813Y | E839D | WT | E540Q, N304S, T260S, A167G, R92K | I207V | I207V | WT | WT | WT | WT | I3T, A42G, K206E, W828S, N865S, R1472Q, F1768I | S362T | WT | I189V | M155T | V239L |
| MYC-19-0101 | V91I, L98S, D243N | WT | K438Q, E839D | WT | E822V, P687L, N304S, R92K | I207V | I207V | WT | WT | G14S | T926P | A42V, T1676S | WT | WT | S284F | WT | WT |
| MYC-19-0102 | S76P, V91I, L98S, T143P, R250K | WT | E839D | WT | N304S, R92K | I207V | I207V | WT | WT | WT | WT | I389V | WT | WT | I189V | V62I | WT |
| MYC-19-0103 | S76P, V91I, L98S, T143P | H58Y | E839D | WT | N462S, N304S, R92K | I207V | I207V | WT | WT | WT | WT | I3T, I25R, I481V, R1472Q, I1512V, Y1731C, I1751V, S1840Y | WT | WT | WT | WT | V239L |
| MYC-19-0104 | S76P, V91I, L98S, T143P, T488R, G493D | H58Y, S844T | E839D, E863D | WT | V477I | I207V | I207V | WT | WT | WT | E1149K | I3T, V645I, G1009D, R1472Q, I1512V, Y1731C, I1751V, L1773I, S1840Y | WT | WT | WT | WT | V239L, S757T |
| MYC-19-0105 | S76P, V91I, L98S, T143P | WT | E839D | F882S | N323Y, N304S | I207V | I207V | WT | WT | WT | WT | I3T, R1472Q, I1512V, A1621T, Y1731C, I1751V, S1840Y | WT | WT | WT | WT | V239L, A942T |
| MYC-19-0106 | S76P, V91I, L98S, T143P, T745A | H58Y, N813Y | E839D | WT | E540Q, N304S, T260S, A167G, R92K | I207V | I207V | WT | WT | WT | WT | I3T, A42G, K206E, N865S, R1472Q, F1768I | S362T | WT | I189V | WT | WT |
| MYC-19-0107 | S76P, V91I, L98S, T143P, R250K | WT | E839D | WT | N304S, G255D, R92K | I207V | I207V | WT | WT | WT | WT | I389V | WT | WT | I189V | V62I | WT |
| MYC-19-0108 | V91I, L98S, D243N | WT | K438Q, E839D | WT | E822V, N304S, R92K | I207V | I207V | WT | WT | G14S | T926P | A42V, T1676S | WT | WT | S284F | WT | WT |
| MYC-19-0109 | V91I, L98S, D243N | WT | K438Q, E839D | WT | E822V, N304S, R92K | I207V | I207V | WT | WT | G14S | T926P | A42V, T1676S | WT | WT | S284F | WT | WT |
| MYC-19-0145 | S76P, V91I, L98S, T143P | WT | E839D | WT | WT | I207V | I207V | WT | WT | WT | S663P | R1039L, N1825S | WT | WT | I189V | WT | P208S, N890I |
| MYC-19-0149 | V91I, L98S, D243N | WT | K438Q, E839D | WT | E822V, N304S, R92K | I207V | I207V | WT | WT | G14S | T926P | A42V, N1290Y, T1676S | WT | WT | S284F | WT | WT |
| MYC-19-0159 | S76P, V91I, L98S, T143P | V914I | E839D | WT | E540Q, N304S, T260S, A167G, R92K | I207V | I207V | R48K | WT | S659* | S663P | I279F, L884V, I1355M, I1469V, R1472Q, I1512V, Y1731C, I1751V | WT | WT | I339M | WT | WT |
| MYC-19-0161 | S76P, V91I, L98S, T143P, E1083G | WT | K438Q, V734I, E839D | G1421S | N304S, R92K | I207V | I207V | WT | WT | D632V | W536* | I389V | H158R | WT | WT | V62I | E231G, L269F |
| MYC-19-0164 | S76P, V91I, L98S, T143P | WT | K438Q, V734I, E839D | G1421S | N304S, R92K | I207V | I207V | WT | WT | WT | WT | I389V | H158R | WT | WT | V62I | E231G, L269F |
| MYC-19-0166 | S76P, V91I, L98S, T143P, W256R | WT | E839D | WT | WT | I207V | I207V | WT | WT | P1024S | WT | R1039L, N1825S | WT | WT | I189V | WT | P208S, N890I |
| MYC-19-0167 | S76P, V91I, L98S, T143P | V914I | E839D | E823D | E540Q, N304S, T260S, A167G, R92K | I207V | I207V | R48K | WT | WT | WT | I279F, L884V, I1355M, I1469V, R1472Q, I1512V, Y1731C, I1751V | WT | WT | I339M | L334P | WT |
| MYC-19-0168 | S76P, V91I, L98S, T143P | H58Y | E839D | N1107K | WT | L60F, I207V | L60F, I207V | WT | WT | WT | E78D, T926P, Ter1898Ter | I3T, R1472Q, I1512V, Y1731C, I1751V, S1840Y | WT | WT | WT | WT | WT |
| MYC-19-0172 | V91I, L98S, V134A | T16I, H58Y | E839D | K1156Q, H1147Q | N304S, R92K | I207V | I207V | WT | WT | WT | WT | T1676S | WT | WT | WT | WT | E478Q |
| MYC-19-0173 | S76P, V91I, L98S, T143P | V914I | E839D | WT | E540Q, N304S, T260S, A167G, R92K | I207V | I207V | R48K | WT | WT | WT | I279F, L884V, I1355M, I1469V, R1472Q, I1512V, Y1731C, I1751V | WT | WT | I339M | WT | WT |
| MYC-19-0174 | V91I, L98S, V134A, S316I | T16I, H58Y, H271R | E839D | K1156Q, H1147Q | N304S, R92K | I207V | I207V | WT | WT | WT | WT | T1676S | WT | WT | WT | WT | E478Q |
| MYC-19-0180 | S76P, V91I, L98S, T143P | H58Y | E839D, T1530K | WT | E540Q, S360N, N304S, Q261del, R92K | I207V | I207V | R48K | WT | WT | S663P, Ter1898Ter | I3T, A42G, K206E, N865S, R1472Q, F1768I | S362T | WT | WT | M155T | V239L |
| MYC-19-0181 | V91I, L98S, D243N, G583S | WT | K438Q, E839D | WT | E822V, P687L, N304S, R92K | I207V | I207V | WT | V155A | G14S | T926P | A42V, T1676S | WT | WT | S284F | WT | WT |
| MYC-19-0184 | S76P, V91I, L98S, T143P | V914I | E839D, V1423A | WT | E540Q, N304S, T260S, A167G, R92K | I207V | I207V | R48K | WT | WT | M603I | I279F, L884V, I1355M, I1469V, R1472Q, I1512V, Y1731C, I1751V | WT | WT | I339M | WT | WT |
| MYC-19-0185 | S76P, V91I, L98S, T143P | H58Y | E839D | WT | E540Q, N304S, T260S, A167G, R92K | I207V | I207V | WT | WT | WT | WT | I3T, R1472Q, I1512V, Y1731C, I1751V, S1840Y | WT | WT | WT | M155T | WT |
| MYC-19-0186 | V27A, S76P, V91I, L98S, T143P | WT | K438Q, V734I, E839D | G1421S | N304S, R92K | I207V | I207V | WT | WT | WT | WT | I389V | H158R | WT | WT | V62I | E231G, L269F |
| MYC-19-0192 | WT | WT | WT | WT | WT | WT | WT | WT | WT | WT | WT | G875S | WT | WT | WT | WT | WT |
| MYC-19-0193 | S76P, V91I, L98S, T143P, G493D | H58Y | E839D, E863D | WT | V477I | I207V | I207V | WT | WT | WT | E1149K | I3T, V645I, G1009D, R1472Q, I1512V, Y1731C, I1751V, L1773I, S1840Y | WT | WT | WT | WT | V239L, S757T |
| MYC-19-0194 | S76P, V91I, L98S, T143P | WT | K438Q, V734I, E839D | G1421S | P643L, N304S, R92K | I207V | I207V | WT | WT | WT | WT | I389V | H158R | G17S | WT | V62I | E231G, L269F |
| MYC-19-0195 | S76P, V91I, L98S, T143P | T16I, H58Y | E839D | H1147Q | A178T | H156Y, I207V | H156Y, I207V | WT | WT | WT | WT | S314F, I960M, D1108E | WT | WT | WT | V62I | T772S |
| MYC-19-0207 | S76P, V91I, L98S, T143P, G493D | H58Y | E839D, E863D | WT | V477I | I207V | I207V | WT | WT | WT | E1149K | I3T, V645I, G1009D, R1472Q, I1512V, Y1731C, I1751V, L1773I, S1840Y | WT | WT | WT | WT | V239L, S757T |
| MYC-19-0208 | S76P, V91I, L98S, T143P | WT | E839D | WT | WT | I207V | I207V | WT | WT | P1024S | WT | R1039L, N1825S | WT | WT | I189V | WT | P208S, N890I |
| MYC-19-0209 | S76P, V91I, L98S, T143P | H58Y, N813Y | E839D | WT | E540Q, N304S, T260S, A167G, R92K | I207V | I207V | WT | WT | WT | WT | I3T, A42G, K206E, N865S, R1472Q, F1768I | S362T | WT | I189V | M155T | V239L |
| MYC-19-0210 | S76P, V91I, L98S, T143P | WT | K438Q, V734I, E839D | G1421S | N304S, R92K | I207V | I207V | WT | WT | WT | WT | I389V | H158R | WT | WT | V62I | E231G, L269F |
| MYC-19-0211 | V91I, L98S, D243N | WT | K438Q, E839D | WT | E822V, P687L, N304S, R92K | I207V | I207V | WT | WT | G14S | T926P | A42V, T1676S | WT | WT | S284F | WT | WT |
| MYC-19-0213 | S76P, V91I, L98S, L139I, T143P | WT | E839D | WT | A178T | I207V | I207V | WT | WT | WT | F384Y | R672K | WT | WT | A55T | WT | WT |
| MYC-19-0215 | S76P, V91I, L98S, T143P | D445N | E839D | G714N | N304S, R92K | I207V | I207V | WT | P424Q | WT | WT | I3T, I481V, R1472Q, I1512V, Y1731C, I1751V, S1840Y | WT | WT | WT | WT | WT |
| MYC-19-0217 | S76P, V91I, L98S, T143P | WT | E839D | WT | WT | I207V | I207V | WT | WT | WT | WT | R1039L, N1825S | WT | WT | I189V | WT | T164K, P208S, N890I |
| MYC-19-0220 | S76P, V91I, L98S, T143P | WT | E839D | WT | WT | I207V | I207V | WT | WT | P1024S | WT | R1039L, N1825S | WT | WT | I189V | WT | P208S, N890I |
| MYC-19-0221 | S76P, V91I, L98S, T143P | WT | K438Q, V734I, E839D | G1421S | N304S, R92K | I207V | I207V | WT | WT | WT | WT | I389V | H158R | WT | WT | V62I | E231G, L269F |
| MYC-19-0227 | S76P, V91I, L98S, T143P | H58Y | E839D | WT | A178T | I207V | I207V | D172Y | WT | WT | WT | I3T, L884V, I1355M, R1472Q, I1512V, Y1731C, I1751V | WT | WT | WT | WT | WT |
| MYC-19-0229 | S76P, V91I, L98S, T143P | WT | E839D | WT | N304S | I207V | I207V | WT | WT | WT | WT | I3T, R1472Q, I1512V, A1621T, Y1731C, I1751V, S1840Y | WT | WT | WT | WT | V239L, A942T |
| MYC-19-0231 | S76P, V91I, L98S, T143P | WT | E839D | WT | WT | I207V | I207V | WT | WT | WT | WT | R1039L, N1825S | WT | WT | I189V | WT | P208S, N890I |
| MYC-19-0232 | S76P, V91I, L98S, T143P | V914I | E839D | WT | E540Q, N304S, T260S, A167G, R92K | I207V | I207V | R48K | WT | WT | WT | I279F, L884V, I1355M, I1469V, R1472Q, I1512V, Y1731C, I1751V | WT | WT | I339M | WT | WT |
| MYC-19-0233 | S76P, V91I, L98S, T143P | WT | E839D | WT | N304S | I207V | I207V | WT | WT | WT | S144F | I3T, R1472Q, I1512V, A1621T, Y1731C, I1751V, S1840Y | WT | WT | WT | WT | V239L, A942T |
| MYC-19-0234 | S76P, V91I, L98S, T143P | D445N | E839D | WT | E540Q, N304S, T260S, R92K | L175I, I207V | L175I, I207V | WT | WT | WT | N23S, D155N | WT | WT | WT | I189V | P148S | WT |
| MYC-19-0235 | S76P, V91I, L98S, T143P | WT | K438Q, V734I, E839D | G1421S | N304S, R92K | I207V | I207V | WT | WT | WT | WT | I389V | H158R | WT | WT | V62I | E231G, L269F |
| MYC-19-0236 | S76P, V91I, L98S, T143P | V914I | E839D | WT | E540Q, N304S, T260S, A167G, R92K | I207V | I207V | R48K | WT | WT | WT | I279F, L884V, I1355M, I1469V, R1472Q, I1512V, Y1731C, I1751V | WT | WT | I339M | WT | WT |
| MYC-19-0237 | S76P, V91I, L98S, T143P | V914I | E839D | WT | E540Q, N304S, T260S, A167G, R92K | I207V | I207V | R48K | WT | WT | WT | I279F, L884V, I1355M, I1469V, R1472Q, I1512V, Y1731C, I1751V | WT | WT | I339M | WT | WT |
| MYC-19-0238 | S76P, V91I, L98S, T143P, I207T, G493D | H58Y, A1120T | E839D, E863D | WT | V477I | I207V | I207V | WT | WT | WT | E1149K | I3T, V645I, G1009D, R1472Q, I1512V, Y1731C, I1751V, L1773I, S1840Y | WT | WT | WT | WT | V239L, S757T |
| MYC-19-0239 | S76P, V91I, L98S, T143P, S651N | H58Y, N813Y | F512C, E839D | WT | N304S, R92K | I207V | I207V | WT | WT | I962L | Ter1898Ter | L884V, E1111G, R1472Q, I1512V, G1515D, Y1731C, I1751V | WT | WT | WT | WT | V239L, I844S |
| MYC-19-0240 | S76P, V91I, L98S, T143P | WT | K438Q, V734I, E839D | G1421S | N304S, R92K | I207V | I207V | WT | WT | WT | WT | I389V | H158R | WT | WT | WT | E231G, L269F |
| MYC-19-0241 | S76P, V91I, L98S, T143P | H58Y | K402N, E839D, T1530K | WT | E540Q, S360N, N304S, R92K | I207V | I207V | R48K | WT | WT | Ter1898Ter | I3T, A42G, K206E, S599F, N865S, R1472Q, F1768I | S362T | WT | WT | WT | V239L |
| MYC-19-0266 | S76P, V91I, L98S, T143P | WT | E839D | WT | WT | I207V | I207V | WT | WT | WT | K1357E | R1039L, N1825S | WT | WT | I189V | WT | P208S, N890I |
| MYC-19-0267 | S76P, V91I, L98S, T143P | K392R | E839D | WT | N304S | I207V | I207V | WT | WT | WT | WT | I3T, R1472Q, I1512V, A1621T, Y1731C, I1751V, S1840Y | WT | WT | WT | WT | V239L, A942T |
| MYC-19-0268 | S76P, V91I, L98S, T143P | WT | K438Q, V734I, E839D | G1421S | N304S, R92K | I207V | I207V | WT | WT | WT | WT | I389V | H158R | WT | WT | V62I | E231G, L269F |
| MYC-19-0269 | S76P, V91I, L98S, T143P | WT | E839D, P1273T | WT | WT | I207V | I207V | WT | WT | WT | F659del | R1039L, N1825S | WT | WT | I189V | G59R | P208S, N890I |
| MYC-19-0270 | V91I, L98S, D243N | WT | K438Q, Q503*, E839D | WT | E822V, N304S, R92K | I207V | I207V | WT | WT | G14S | T926P | A42V, T1676S | WT | WT | S284F | WT | WT |
| MYC-19-0271 | S76P, RV90II, L98S, T143P, S391L | V35E, F551L, L554F, F774fs | K438Q, V734I, E839D | G1421S | N304S, R92K | I207V | I207V | WT | Y141F | L523F | S663F | I389V | H158R | WT | WT | V62I | E231G, L269F |
| MYC-19-0272 | S76P, V91I, L98S, T143P | WT | K438Q, V734I, E839D | G1421S | N304S, R92K | I207V | I207V | WT | WT | WT | WT | I389V | H158R | WT | WT | V62I | E231G, L269F |
| MYC-19-0273 | S76P, V91I, L98S, T143P, G493D, G611V, D876N | H58Y | E839D, E863D | WT | V477I | I207V | I207V | WT | WT | WT | W1489C | I3T, V645I, G1009D, R1472Q, I1512V, Y1731C, I1751V, L1773I, S1840Y | WT | WT | WT | WT | V239L, S757T |
| MYC-19-0274 | S76P, V91I, L98S, T143P, P235L | H58Y, D445N, S815T | E839D | F773S | WT | I207V | I207V | WT | WT | WT | WT | WT | I103V | WT | WT | WT | WT |
| MYC-19-0275 | V91I, L98S, D243N, S343P | WT | K438Q, E839D | WT | E822V, N304S, R92K | I207V | I207V | WT | WT | G14S | T926P | A42V, T1676S | WT | WT | S284F | WT | WT |
| MYC-19-0289 | S76P, V91I, L98S, T143P | WT | E839D | WT | N323Y, N304S | I207V | I207V | P289Q | WT | WT | T1712I | I3T, R1472Q, I1512V, A1621T, Y1731C, I1751V, S1840Y | WT | WT | WT | WT | V239L, A942T |
| MYC-19-0291 | V91I, L98S, D243N | WT | K438Q, E839D | P1104H | E822V, N304S, R92K | I207V | I207V | WT | WT | G14S | T926P | A42V, T1676S | WT | WT | S284F | WT | WT |
| MYC-19-0292 | S76P, V91I, L98S, T143P | T1448I | E839D | WT | N304S | I207V | I207V | WT | WT | N159H | WT | I3T, R1472Q, I1512V, A1621T, Y1731C, I1751V, S1840Y | WT | WT | WT | WT | V239L, A942T |
| MYC-19-0297 | S76P, V91I, L98S, T143P, D876N | WT | E839D, G872S | WT | N304S, T260_Q261insQQ | I207V | I207V | WT | WT | WT | WT | I3T, Q1142R, R1472Q, I1512V, A1621T, Y1731C, I1751V, S1840Y | WT | WT | WT | WT | V239L, A942T |
| MYC-19-0298 | V91I, L98S, D243N | WT | K438Q, E839D | WT | E822V, P687L, N304S, Q261del, R92K | I207V | I207V | WT | WT | G14S | T926P | A42V, T1676S | WT | WT | S284F | WT | WT |
| MYC-19-0303 | V91I, L98S, D243N | WT | K438Q, E839D | WT | E822V, N304S, R92K | I207V | I207V | WT | WT | G14S | V621L, T926P | A42V, T1676S | WT | WT | S284F | WT | WT |
| MYC-19-0304 | V91I, L98S, D243N | WT | K438Q, E839D | WT | E822V, N304S, R92K | I207V | I207V | WT | WT | G14S | T926P, L1772F | A42V, A151fs, T1676S | WT | WT | S284F | WT | E7K |
| MYC-19-0306 | S76P, V91I, L98S, T143P | V914I | E839D | WT | E540Q, N304S, T260S, A167G, R92K | I207V | I207V | R48K | WT | WT | WT | I279F, G464R, E713D, L884V, I1355M, I1469V, R1472Q, I1512V, Y1731C, I1751V | WT | WT | I339M | WT | WT |
| MYC-19-0308 | S76P, V91I, L98S, T143P | WT | E839D | WT | N323Y, N304S | I207V | I207V | WT | WT | WT | WT | I3T, R1472Q, I1512V, A1621T, Y1731C, I1751V, S1840Y | WT | WT | WT | WT | V239L, A942T |
| MYC-19-0315 | S76P, V91I, L98S, T143P | WT | E839D, E863D | WT | E540Q, N304S, T260S, A167G, R92K | I207V | I207V | R48K | WT | WT | R1378S, Ter1898Ter | I3T, R1472Q, I1512V, Y1731C, I1751V, R1761M, S1840Y | WT | WT | I189V | WT | WT |
| MYC-19-0316 | S76P, V91I, L98S, T143P | H58Y, D445N, S815T | E839D | F773S | WT | I207V | I207V | WT | WT | F625S | WT | WT | I103V | WT | WT | WT | WT |
| MYC-19-0317 | V91I, L98S, D243N, G583S | WT | K438Q, E839D | WT | E822V, P687L, N304S, R92K | I207V | I207V | WT | V155A | G14S | T926P | A42V, T1676S | WT | WT | S284F | WT | WT |
| MYC-19-0318 | V91I, L98S, D243N, E340K | WT | K438Q, E839D | WT | E822V, P687L, N304S, R92K | I207V | I207V | V273I | WT | G14S | T926P | A42V, T1676S | WT | WT | S284F | WT | WT |
| MYC-19-0319 | S76P, V91I, L98S, T143P, D876Y | H58Y | E839D | WT | WT | L60F, I207V | L60F, I207V | WT | WT | WT | E78D, T926P, Ter1898Ter | I3T, R1472Q, I1512V, Y1731C, I1751V, S1840Y | WT | Q132H | WT | WT | WT |
| MYC-19-0320 | S76P, V91I, L98S, T143P, D876N | H58Y | E839D | WT | WT | L60F, I207V | L60F, I207V | WT | WT | WT | E78D, T926P, Ter1898Ter | I3T, R1472Q, I1512V, Y1731C, I1751V, S1840Y | WT | Q132H | WT | WT | WT |
| MYC-19-0321 | S76P, V91I, L98S, T143P | WT | E839D | WT | N304S | I207V | I207V | WT | WT | WT | S144F | I3T, R1472Q, I1512V, A1621T, Y1731C, I1751V, S1840Y | WT | WT | WT | WT | V239L, A942T |
| MYC-19-0322 | S76P, V91I, L98S, L139I, T143P | WT | E839D | WT | A178T | I207V | I207V | WT | WT | WT | F384Y | R672K | WT | WT | A55T | WT | WT |
| MYC-19-0323 | S76P, V91I, L98S, T143P | H58Y | E839D | K830N | E540Q, N304S, T260S, A167G | I207V | I207V | WT | WT | WT | WT | WT | I103V, V71F | WT | WT | WT | F666I |
| MYC-19-0324 | V91I, L98S, D243N, D1089Y | WT | K438Q, E839D | P1104H | E822V, N304S, R92K | I207V | I207V | WT | WT | G14S | T926P | A42V, T1676S | WT | WT | S284F | WT | WT |
| MYC-19-0325 | S76P, V91I, L98S, T143P, T745A, L1081F | WT | K438Q, V734I, E839D | WT | N304S, R92K | I207V | I207V | WT | WT | WT | E836A, A982S, I1781M | I3T, E40V, I109V, K187N, I190L, G280E, M429I, D851H, S854R, I1512V, Y1731C, I1751V | WT | WT | S284F | WT | L810H |
| MYC-19-0326 | V91I, L98S, D243N, G1099D | WT | K438Q, E839D | WT | E822V, N304S, R92K | I207V | I207V | WT | WT | G14S | T926P, L1772F | A42V, A151fs, T1676S | WT | WT | S284F | WT | E7K |
| MYC-19-0327 | S76P, V91I, L98S, T143P | V914I | E839D | WT | E540Q, N304S, T260S, A167G, R92K | I207V | I207V | R48K | L20P | WT | WT | I279F, L884V, I1355M, I1469V, R1472Q, I1512V, Y1731C, I1751V | WT | WT | I339M | WT | WT |
| MYC-19-0328 | S76P, V91I, L98S, T143P | H58Y | E839D, T1530K | WT | E540Q, S360N, N304S, R92K | I207V | I207V | R48K | WT | WT | Ter1898Ter | I3T, A42G, K206E, N865S, R1472Q, F1768I | S362T | WT | WT | M155T | V239L |
| MYC-19-0329 | S76P, V91I, L98S, T143P | H58Y, D445N, S815T | E839D | F773S | WT | I207V | I207V | WT | WT | WT | WT | WT | I103V | WT | WT | WT | WT |
| MYC-19-0330 | S76P, V91I, L98S, Y124S, T143P | WT | E839D | WT | WT | I207V | I207V | WT | WT | WT | WT | R1039L, N1825S | WT | WT | I189V | WT | P208S, N890I |
| MYC-19-0331 | S76P, V91I, L98S, T143P | H58Y | E839D, T1530K | WT | E540Q, S360N, N304S, R92K | I207V | I207V | R48K | WT | WT | Ter1898Ter | I3T, A42G, K206E, N865S, R1472Q, F1768I | S362T | WT | WT | M155T | V239L |
| MYC-19-0332 | S76P, V91I, L98S, T143P, R250K, S942F | WT | E839D | WT | N304S, Q261del, R92K | I207V | I207V | WT | WT | WT | WT | I389V | WT | WT | I189V | V62I | WT |
| MYC-19-0333 | S76P, V91I, L98S, T143P | H1098Y | K438Q, V734I, E839D | G1421S | L528I, N304S, R92K | I207V | I207V | WT | WT | WT | WT | I389V | H158R | WT | WT | V62I | E231G, L269F |
| MYC-19-0334 | V91I, L98S, D243N, G583S | WT | K438Q, E839D | WT | E822V, P687L, N304S, R92K | I207V | I207V | WT | V155A | G14S | T926P | A42V, T1676S | WT | WT | S284F | WT | WT |
| MYC-19-0335 | V91I, L98S, D243N, A693E | WT | K438Q, E839D | WT | E822V, N304S, Q261del, R92K | I207V | I207V | WT | WT | G14S | T926P | A42V, T1676S | WT | WT | S284F | WT | WT |
| MYC-19-0336 | S76P, V91I, L98S, T143P, K274E, F613del | WT | E839D | WT | N304S | I207V | I207V | WT | WT | WT | WT | I3T, R1472Q, I1512V, A1621T, Y1731C, I1751V, S1840Y | WT | WT | WT | WT | V239L, A942T |
| MYC-19-0337 | S76P, V91I, L98S, T143P | WT | E839D | WT | N323Y, N304S | I207V | I207V | WT | WT | WT | WT | I3T, Q153R, R1472Q, I1512V, A1621T, Y1731C, I1751V, S1840Y | WT | WT | WT | WT | V239L, A942T |
| MYC-19-0338 | V91I, L98S, D243N, L291F | WT | K438Q, E839D | P1104H | E822V, N304S, R92K | I207V | I207V | WT | WT | G14S | T926P | A42V, T1676S | WT | WT | S284F | WT | WT |
| MYC-19-0339 | S76P, V91I, L98S, T143P, G346D | WT | K438Q, V734I, E839D | G1421S | N304S, R92K | I207V | I207V | WT | WT | WT | WT | I389V | H158R | WT | WT | V62I | E231G, L269F |
| MYC-19-0340 | S76P, V91I, L98S, L139I, T143P, T360I | WT | E839D | WT | A178T | I207V | I207V | WT | WT | WT | F384Y | R672K | WT | WT | A55T | WT | WT |
| MYC-19-0341 | V91I, L98S, D243N, L931F | WT | K438Q, E839D | P1104H | E822V, N304S, R92K | I207V | I207V | WT | WT | G14S | T926P | A42V, T1676S | WT | WT | S284F | WT | WT |
| MYC-19-0342 | S76P, V91I, L98S, T143P, T1080A | WT | E839D | WT | N304S | I207V | I207V | WT | WT | N159H | WT | I3T, R1472Q, I1512V, A1621T, Y1731C, I1751V, S1840Y | WT | WT | WT | WT | V239L, A942T |
| MYC-19-0343 | V91I, L98S, D243N, C350R | WT | K438Q, E839D | WT | E822V, N304S, R92K | I207V | I207V | WT | WT | G14S | T926P | A42V, T1676S | WT | WT | S284F | WT | WT |
| MYC-19-0344 | V91I, L98S, D243N | WT | K438Q, E839D | P1104H | E822V, N304S, R92K | I207V | I207V | WT | WT | G14S | T926P | A42V, T1676S | WT | WT | S284F | WT | WT |
| MYC-19-0345 | S76P, V91I, L98S, T143P, G189V, G1079R | H58Y | E839D | WT | E540Q, N304S, T260S, R92K | I207V | I207V | G147A, I323L | WT | WT | WT | A1110T | WT | WT | I189V | WT | WT |
| MYC-19-0346 | S76P, V91I, L98S, T143P, R761S | H58Y | E839D | G624R | WT | L60F, I207V | L60F, I207V | WT | WT | WT | E78D, T926P, Ter1898Ter | I3T, R1472Q, I1512V, Y1731C, I1751V, S1840Y | WT | Q132H | WT | WT | WT |
| MYC-19-0386 | V91I, L98S, D243N | WT | K438Q, E839D | P1104H | E822V, N304S, R92K | I207V | I207V | WT | WT | G14S | S663P, T926P | A42V, T1676S | WT | WT | S284F | WT | WT |

**Supplementary Table S5. Genes with copy number variations detected exclusively in fluconazole-resistant *Candida glabrata* isolates.**

| **Chr** | **Gene^a^** | **Gene Name** | **CNV Status** | **Copy Number** | **Isolate(s)** | **Description^b^** |
| --- | --- | --- | --- | --- | --- | --- |
| A | CAGL0A02046g |  | increase | 2.01 | MYC-19-0008 | Ortholog(s) have ubiquitin protein ligase binding activity and role in cellular response to glucose stimulus, positive regulation of receptor internalization, response to xenobiotic stimulus, ubiquitin-dependent endocytosis |
| F | CAGL0F08855g |  | increase | 2.09-2.46 | MYC-19-0008, MYC-19-0088 | Ortholog(s) have C-acetyltransferase activity |
| F | CAGL0F08877g |  | increase | 2.09 | MYC-19-0008 | Ortholog of *S. cerevisiae*: YGR016W and *Saccharomyces cerevisiae* S288C: YGR016W |
| F | CAGL0F08899r |  | increase | 2.09 | MYC-19-0008 | tRNA-Leu, predicted by tRNAscan-SE; CAA anticodon |
| H | CAGL0H03289g |  | increase | 2.06 | MYC-19-0008 | Has domain(s) with predicted K48-linked deubiquitinase activity, cysteine-type deubiquitinase activity |
| K | CAGL0K09988g |  | increase | 2.06 | MYC-19-0008 | Ortholog(s) have role in vesicle-mediated transport and Golgi membrane localization |
| K | CAGL0K10098g |  | increase | 2.14 | MYC-19-0008 | Ortholog(s) have role in nucleotide-excision repair, transcription by RNA polymerase II, transcription initiation at RNA polymerase II promoter |
| K | CAGL0K10120g |  | increase | 2.14 | MYC-19-0008 | Ortholog(s) have unfolded protein binding activity, role in mitochondrial cytochrome c oxidase assembly and mitochondrial inner membrane localization |
| K | CAGL0K11132g |  | increase | 2.02 | MYC-19-0008 | Ortholog(s) have mRNA binding activity, role in mRNA splicing, via spliceosome and U1 snRNP, U2-type prespliceosome, commitment complex localization |
| K | CAGL0K12430g | *STE2* | increase | 2.38 | MYC-19-0008 | Ortholog(s) have mating-type P-factor pheromone receptor activity, mating-type alpha-factor pheromone receptor activity |
| K | CAGL0K12452g |  | increase | 2.38 | MYC-19-0008 | Ortholog(s) have GTPase activator activity, role in regulation of Rho protein signal transduction, septum digestion after cytokinesis, vesicle-mediated transport and cytoplasm, endoplasmic reticulum, peroxisome localization |
| K | CAGL0K12606g |  | increase | 2.3 | MYC-19-0008 | Ortholog(s) have protein kinase activator activity |
| L | CAGL0L06952g | *TIF34* | increase | 2.05 | MYC-19-0008 | Translation initiation factor eIF3, p39 subunit; protein abundance decreased in ace2 mutant cells |
| L | CAGL0L06974g |  | increase | 2.05 | MYC-19-0008 | Has domain(s) with predicted hydrolase activity |
| L | CAGL0L11330g |  | increase | 2.29 | MYC-19-0008 | Ortholog(s) have role in regulation of translational termination |
| B | **CAGL0B02882g** | *BMT2* | decrease | 0 | MYC-19-0025 | Beta mannosyltransferase; transmembrane domain protein similar to *C. albicans* WRY family; gene is downregulated in azole-resistant strain |
| A | CAGL0A02794g |  | increase | 2.13 | MYC-19-0082 | Ortholog(s) have inositol phosphoceramide synthase regulator activity, role in inositol phosphoceramide metabolic process and Golgi membrane, inositol phosphoceramide synthase complex localization |
| A | CAGL0A03344g |  | increase | 2.01 | MYC-19-0082 | Ortholog(s) have ubiquitin-protein transferase activity and role in cellular response to amino acid stimulus, proteasome-mediated ubiquitin-dependent protein catabolic process, ubiquitin-dependent protein catabolic process |
| A | CAGL0A03630g |  | increase | 2.13 | MYC-19-0082 | Ortholog(s) have RNA polymerase III general transcription initiation factor activity, chromatin insulator sequence binding activity and role in transcription initiation at RNA polymerase III promoter |
| A | CAGL0A03696g | *GAL83* | increase | 2.28 | MYC-19-0082 | GAL83; Ortholog(s) have AMP-activated protein kinase activity, enzyme-substrate adaptor activity |
| A | CAGL0A04587g |  | increase | 2.06 | MYC-19-0088 | Ortholog(s) have dol-P-Man:Man(5)GlcNAc(2)-PP-Dol alpha-1,3-mannosyltransferase activity |
| F | CAGL0F08833g |  | increase | 2.25 | MYC-19-0088 | Putative adhesin-like protein |
| A | CAGL0A04477g |  | increase | 2.08-2.25 | MYC-19-0092, MYC-19-0149 | Ortholog(s) have cysteine-type deubiquitinase activity, metal-dependent deubiquitinase activity and role in endocytosis, free ubiquitin chain depolymerization, protein K11-linked deubiquitination, protein K48-linked deubiquitination |
| E | CAGL0E01881g | *YPS11* | increase | 4.07 | MYC-19-0092 | Putative aspartic protease: member of a YPS gene cluster that is required for virulence in mice; induced in response to low pH and high temperature |
| E | CAGL0E02189g |  | increase | 4.68 | MYC-19-0092 | Ortholog(s) have palmitoyltransferase activity, role in protein palmitoylation, protein targeting to membrane and endoplasmic reticulum palmitoyltransferase complex localization |
| E | CAGL0E02211g |  | increase | 4.19 | MYC-19-0092 | Protein of unknown function |
| E | CAGL0E02233g |  | increase | 4.19 | MYC-19-0092 | Has domain(s) with predicted protein kinase binding activity and role in regulation of cyclin-dependent protein serine/threonine kinase activity |
| E | CAGL0E02255g |  | increase | 2.37 | MYC-19-0092 | Ortholog(s) have role in fungal-type cell wall organization and plasma membrane localization |
| E | CAGL0E02277r |  | increase | 3.37 | MYC-19-0092 | tRNA-Gly, predicted by tRNAscan-SE; UCC anticodon |
| E | CAGL0E02365g | *MDM12* | increase | 3.91 | MYC-19-0092 | Putative subunit of the mitochondrial ERMES complex that acts in mitochondrion organization and tethering mitochondria to the ER; null mutants show decreased vegetative growth on both glucose and glycerol media |
| F | CAGL0F05357g | *ZCF11* | increase | 2.02 | MYC-19-0092 | Zinc cluster transcription factor; involved in regulation of genes related to meiosis, though meiosis has not been observed; essential gene; appears at least partially functionally homologous to *S. cerevisiae UME6* |
| F | **CAGL0F08041g** | ***PFK1*** | increase | 2.04-2.11 | MYC-19-0092, MYC-19-0149 | Putative phosphofructokinase, alpha subunit; increased protein abundance in azole resistant strain |
| G | CAGL0G01166g |  | increase | 2.21 | MYC-19-0092 | Protein of unknown function |
| G | CAGL0G05588g |  | increase | 2.03-2.36 | MYC-19-0092, MYC-19-0149 | Ortholog(s) have single-stranded telomeric DNA binding, telomerase inhibitor activity, translation elongation factor binding activity |
| G | CAGL0G07447g |  | increase | 2.11 | MYC-19-0092 | Ortholog of *S. cerevisiae*: MRX4 and *S. cerevisiae* S288C: YPL168W |
| H | CAGL0H00682g |  | increase | 2.17 | MYC-19-0092 | Has domain(s) with predicted Glc3Man9GlcNAc2 oligosaccharide glucosidase activity and role in carbohydrate metabolic process, oligosaccharide metabolic process |
| K | CAGL0K00605g |  | increase | 2.11 | MYC-19-0092 | Ortholog(s) have ATP binding, ATPase, DNA replication origin binding, GTP binding, GTPase activity, chromatin binding, cyclin-dependent protein serine/threonine kinase inhibitor activity |
| K | **CAGL0K05841g** | ***HAP1B*** | increase | 2.18 | MYC-19-0092 | Zinc cluster transcription factor required for regulation of ERG genes in response to azoles; appears partially redundant with HAP1A; null mutant shows increased susceptibility to fluconazole |
| K | CAGL0K06083g |  | increase | 2.33 | MYC-19-0092 | Ortholog(s) have DNA end binding activity and role in double-strand break repair via nonhomologous end joining, double-strand break repair via single-strand annealing, homologous recombination, positive regulation of DNA ligase activity |
| K | CAGL0K06127g |  | increase | 2.23 | MYC-19-0092 | Ortholog(s) have role in reciprocal meiotic recombination, replication-born double-strand break repair via sister chromatid exchange, synaptonemal complex organization and nucleus, synaptonemal complex localization |
| K | CAGL0K06149g |  | increase | 2.23 | MYC-19-0092 | Ortholog(s) have role in ribosomal small subunit assembly |
| K | CAGL0K08536g | *APE1* | increase | 2 | MYC-19-0092 | Vacuolar aminopeptidase I |
| K | CAGL0K08558g |  | increase | 2 | MYC-19-0092 | Ortholog(s) have glutamine-fructose-6-phosphate transaminase (isomerizing) activity and role in fungal-type cell wall chitin biosynthetic process |
| M | CAGL0M05621g |  | increase | 2.04-2.2 | MYC-19-0092, MYC-19-0273 | Ortholog(s) have inositol phosphorylceramide mannosyltransferase activity and role in glycosphingolipid biosynthetic process, mannosyl-inositol phosphorylceramide biosynthetic process, sphingolipid biosynthetic process |
| A | CAGL0A03388g |  | increase | 2.19 | MYC-19-0093 | Ortholog(s) have RNA binding, structural constituent of ribosome activity, role in cytoplasmic translation and cytosolic large ribosomal subunit localization |
| A | CAGL0A03432g |  | increase | 2.27 | MYC-19-0093 | Ortholog(s) have ATP-dependent activity, acting on DNA, Y-form DNA binding, four-way junction DNA binding, four-way junction helicase activity, ubiquitin protein ligase activity |
| H | CAGL0H09284r |  | increase | 2.18-2.36 | MYC-19-0093, MYC-19-0343 | tT(AGU)7; tRNA-Thr, predicted by tRNAscan-SE; AGU anticodon |
| K | CAGL0K06875g |  | increase | 2.1-2.51 | MYC-19-0093, MYC-19-0149 | Ortholog(s) have 3'-deoxyribose phosphate lyase activity, 3'-tyrosyl-DNA phosphodiesterase activity, 5'-tyrosyl-DNA phosphodiesterase activity, role in DNA repair, base-excision repair and mitochondrion, nucleus localization |
| K | CAGL0K07656g | *FRS2* | increase | 2.03 | MYC-19-0093 | Putative cytosolic phenylalanine?tRNA ligase, beta chain |
| F | CAGL0F05159g |  | increase | 2 | MYC-19-0094 | Ortholog(s) have histone binding activity, role in chromatin remodeling and Swr1 complex localization |
| F | CAGL0F05665g |  | increase | 2.19-2.25 | MYC-19-0094, MYC-19-0345 | Ortholog(s) have SNARE binding, syntaxin binding activity |
| G | CAGL0G06380g |  | increase | 2.29 | MYC-19-0094 | Ortholog(s) have NuA4 histone acetyltransferase complex binding activity, role in aerobic respiration and nucleus localization |
| I | CAGL0I04576g |  | increase | 2.01 | MYC-19-0094 | Ortholog(s) have anaphase-promoting complex localization |
| I | CAGL0I04598g |  | increase | 2.01 | MYC-19-0094 | Ortholog(s) have role in mitochondrial respiratory chain complex II assembly and mitochondrial inner membrane localization |
| A | CAGL0A03300g |  | increase | 2.05 | MYC-19-0149 | Ortholog(s) have mRNA (N6-adenosine)-methyltransferase activity and role in mRNA methylation, meiotic DNA replication initiation, meiotic cell cycle, negative regulation of pseudohyphal growth |
| C | CAGL0C01221g |  | increase | 2.15 | MYC-19-0149 | Ortholog(s) have guanyl-nucleotide exchange factor activity, translation initiation factor activity, role in cytoplasmic translational initiation and eukaryotic translation initiation factor 2B complex localization |
| C | CAGL0C01639g |  | increase | 2.19 | MYC-19-0149 | Ortholog(s) have snoRNA binding activity |
| C | CAGL0C01661g |  | increase | 2.19 | MYC-19-0149 | Ortholog(s) have diphthine methylesterase activity, role in endocytic recycling, protein histidyl modification to diphthamide and cytoplasm, endosome localization |
| C | CAGL0C01727g |  | increase | 2.07 | MYC-19-0149 | Ortholog(s) have UDP-N-acetylglucosamine-dolichyl-phosphate N-acetylglucosaminephosphotransferase activity and role in aerobic respiration, dolichol-linked oligosaccharide biosynthetic process, protein N-linked glycosylation |
| C | CAGL0C01749g |  | increase | 2.07 | MYC-19-0149 | Ortholog(s) have 5'-deoxynucleotidase activity, GMP 5'-nucleotidase activity and role in deoxyribonucleoside monophosphate catabolic process |
| C | CAGL0C02387g |  | increase | 2.16 | MYC-19-0149 | Ortholog of *S. cerevisiae*: YER034W and *S. cerevisiae* S288C: YER034W |
| C | CAGL0C03333g | *FRE6* | increase | 2.28 | MYC-19-0149 | Protein involved in reductive iron uptake; orthologs have ferric-chelate reductase activity; null mutant shows decreased adhesion to ovary epithelial cells but increased survival in mouse liver |
| C | CAGL0C03399g | *POP2* | increase | 2.41 | MYC-19-0149 | Ortholog(s) have 3'-5'-RNA exonuclease activity and role in nuclear-transcribed mRNA poly(A) tail shortening, positive regulation of transcription elongation by RNA polymerase II, transcription elongation by RNA polymerase II |
| C | CAGL0C03443g | *LYS9* | increase | 2.15 | MYC-19-0149 | Putative saccharopine dehydrogenase |
| C | CAGL0C03465g |  | increase | 2.15 | MYC-19-0149 | Ortholog(s) have role in ascospore-type prospore membrane formation, membrane fusion, vesicle docking involved in exocytosis |
| D | CAGL0D03740g |  | increase | 2.01 | MYC-19-0149 | Ortholog(s) have RNA binding, ribonuclease MRP activity, ribonuclease P activity |
| F | CAGL0F00429g |  | increase | 2.07 | MYC-19-0149 | Ortholog(s) have GTP diphosphatase activity, ITP diphosphatase activity, UTP diphosphatase activity, XTP diphosphatase activity, dATP diphosphatase activity and dCTP diphosphatase activity |
| F | CAGL0F00451g |  | increase | 2.07 | MYC-19-0149 | Ortholog(s) have DNA clamp loader activity, chromatin binding activity |
| F | CAGL0F00627g |  | increase | 2.05 | MYC-19-0149 | Ortholog(s) have RNA binding activity, role in intracellular copper ion homeostasis, regulation of translation and cytoplasmic stress granule localization |
| F | CAGL0F04433g | *URA7* | increase | 2.25 | MYC-19-0149 | CTP synthase |
| F | CAGL0F04807g |  | increase | 2.12 | MYC-19-0149 | Ortholog(s) have mitochondrial outer membrane, mitochondrion localization |
| F | CAGL0F04829g |  | increase | 2.12 | MYC-19-0149 | Ortholog(s) have role in endocytosis, negative regulation of Arp2/3 complex-mediated actin nucleation and actin cortical patch, cell cortex, cytoplasm, cytosol localization |
| F | CAGL0F04851g |  | increase | 2.07 | MYC-19-0149 | Ortholog(s) have role in aerobic respiration, mRNA metabolic process |
| F | CAGL0F04873g |  | increase | 2.51 | MYC-19-0149 | Ortholog(s) have glucosidase activity and role in (1->6)-beta-D-glucan biosynthetic process, fungal-type cell wall organization |
| G | CAGL0G05401g |  | increase | 2.14 | MYC-19-0149 | Ortholog(s) have mRNA binding activity and role in invasive growth in response to glucose limitation, mRNA destabilization, pseudohyphal growth, regulation of mRNA catabolic process, traversing start control point of mitotic cell cycle |
| G | CAGL0G05522g |  | increase | 2.17 | MYC-19-0149 | Protein of unknown function |
| G | CAGL0G06160g |  | increase | 2.11 | MYC-19-0149 | Protein of unknown function |
| G | CAGL0G06182g |  | increase | 2.11 | MYC-19-0149 | Ortholog of *S. cerevisiae*: YHR131C, *C. albicans* SC5314: C5_03510C_A, *C. dubliniensis* CD36: Cd36_53260, *C. parapsilosis* CDC317: CPAR2_101770 and *C. auris* B8441: B9J08_004698 |
| G | CAGL0G07425g |  | increase | 2.04 | MYC-19-0149 | Ortholog(s) have AMP deaminase activity and role in guanine salvage, purine nucleotide metabolic process |
| G | CAGL0G08470g |  | increase | 2.03 | MYC-19-0149 | Ortholog(s) have DNA topoisomerase activity, DNA topoisomerase type I (single strand cut, ATP-independent) activity |
| G | CAGL0G08492g |  | increase | 2.03 | MYC-19-0149 | Ortholog(s) have RNA binding, single-stranded DNA binding, telomeric DNA binding activity and role in G-quadruplex DNA formation, regulation of telomere maintenance via telomerase, telomere maintenance, telomere maintenance via telomerase |
| G | CAGL0G08514r |  | increase | 2.01 | MYC-19-0149 | tI(UAU)2; tRNA-Ile, predicted by tRNAscan-SE; UAU anticodon |
| G | CAGL0G08558g |  | increase | 2.01 | MYC-19-0149 | Ortholog(s) have GTP binding, GTPase activity |
| H | CAGL0H02585g |  | increase | 2.05 | MYC-19-0149 | Ortholog(s) have glutamate decarboxylase activity and role in cellular response to oxidative stress, glutamate catabolic process |
| H | CAGL0H04081g | *ERG13* | increase | 2.03 | MYC-19-0149 | 3-hydroxy-3-methylglutaryl coenzyme A synthase; protein abundance increased in ace2 mutant cells |
| H | CAGL0H10186g |  | increase | 2.14 | MYC-19-0149 | Ortholog(s) have cysteine-type deubiquitinase activity and role in negative regulation of gluconeogenesis, proteasome-mediated ubiquitin-dependent protein catabolic process |
| H | CAGL0H10202g |  | increase | 2.14 | MYC-19-0149 | Ortholog(s) have enzyme activator activity, role in 3-keto-sphinganine metabolic process, positive regulation of sphingolipid biosynthetic process and SPOTS complex localization |
| H | CAGL0H10208g |  | increase | 2.14 | MYC-19-0149 | Ortholog(s) have protein serine/threonine kinase activity and role in negative regulation of clathrin-dependent endocytosis |
| H | CAGL0H10516g |  | increase | 2.47 | MYC-19-0149 | Ortholog(s) have enzyme regulator activity and role in mitotic actomyosin contractile ring contraction, mitotic division septum assembly, secondary cell septum biogenesis |
| H | CAGL0H10560g |  | increase | 2.14 | MYC-19-0149 | Ortholog(s) have endoplasmic reticulum localization |
| I | CAGL0I01056g |  | increase | 2.21 | MYC-19-0149 | Protein of unknown function |
| I | CAGL0I01122g | *GRE3* | increase | 2.08 | MYC-19-0149 | Ortholog(s) have D-xylose:NADP reductase activity, alditol:NADP+ 1-oxidoreductase activity, mRNA binding activity |
| I | CAGL0I03960g | *VMA1* | increase | 2.1 | MYC-19-0149 | Putative site-specific DNA endonuclease with autocatalytic protein-splicing activity |
| I | CAGL0I04752g |  | increase | 2.62 | MYC-19-0149 | Ortholog(s) have phosphatidate cytidylyltransferase activity and role in CDP-diacylglycerol biosynthetic process, phosphatidylinositol metabolic process, phosphatidylserine metabolic process |
| I | CAGL0I04774g |  | increase | 2.62 | MYC-19-0149 | Ortholog(s) have calcium-release channel activity, enzyme regulator activity |
| J | CAGL0J00165g |  | increase | 2.02 | MYC-19-0149 | Ortholog(s) have structural constituent of ribosome activity and cytosolic small ribosomal subunit localization |
| J | CAGL0J00781g |  | increase | 2.11 | MYC-19-0149 | Ortholog(s) have phospholipid binding, structural constituent of nuclear pore activity |
| J | CAGL0J05412g |  | increase | 2.04 | MYC-19-0149 | Ortholog(s) have role in ribosomal large subunit assembly, ribosomal subunit export from nucleus and cytoplasm, cytosolic large ribosomal subunit localization |
| K | CAGL0K04499g |  | increase | 2.1 | MYC-19-0149 | Ortholog(s) have phosphoribosylformylglycinamidine synthase activity and role in purine nucleotide biosynthetic process |
| K | CAGL0K06039g | *RED1* | increase | 2.13 | MYC-19-0149 | Ortholog(s) have chromatin DNA binding, structural molecule activity, role in meiotic recombination checkpoint signaling, synaptonemal complex assembly and lateral element localization |
| K | CAGL0K06061g |  | increase | 2.13 | MYC-19-0149 | Ortholog(s) have structural constituent of ribosome activity |
| K | CAGL0K12650g | *TUB2* | increase | 2.03 | MYC-19-0149 | Beta-tubulin; protein abundance decreased in ace2 mutant cells |
| K | CAGL0K12672g |  | increase | 2.03 | MYC-19-0149 | Ortholog(s) have GTPase activity, SNARE binding activity |
| L | CAGL0L11440g |  | increase | 2.15 | MYC-19-0149 | Ortholog(s) have lipid binding, phospholipid binding activity |
| M | CAGL0M00660g |  | increase | 2.05 | MYC-19-0149 | Ortholog(s) have proton-transporting ATPase activity, rotational mechanism activity, role in vacuolar transport and fungal-type vacuole membrane localization |
| M | CAGL0M00682g |  | increase | 2.05 | MYC-19-0149 | Ortholog(s) have N-acetylglucosamine kinase activity |
| M | CAGL0M08250g |  | increase | 2.12 | MYC-19-0149 | Ortholog(s) have zinc ion transmembrane transporter activity, role in intracellular zinc ion homeostasis, zinc ion transport and fungal-type vacuole membrane localization |
| C | CAGL0C01815g |  | increase | 2-2.05 | MYC-19-0161, MYC-19-0341 | Ortholog of *S. cerevisiae*: YBL086C, *C. albicans* SC5314: C7_02530C_A, *C. dubliniensis* CD36: Cd36_72270, *C. parapsilosis* CDC317: CPAR2_702850 and *C. auris* B8441: B9J08_001027 |
| G | CAGL0G06578g |  | increase | 2.02 | MYC-19-0161 | Ortholog(s) have role in cellular response to oxidative stress |
| I | CAGL0I08349g |  | increase | 2.05-2.07 | MYC-19-0161, MYC-19-0342 | Ortholog(s) have ATP binding, RNA polymerase II CTD heptapeptide repeat S5 kinase activity and RNA polymerase II CTD heptapeptide repeat kinase activity |
| K | CAGL0K12034g | *ENA1* | increase | 2.03 | MYC-19-0161 | Na(+)-ATPase with broad substrate specificity; plays a role in sodium detoxification; highly upregulated by increased osmotic pressure or sodium concentration |
| L | CAGL0L09537g |  | increase | 2.04 | MYC-19-0161 | Has domain(s) with predicted FMN binding, oxidoreductase activity |
| C | CAGL0C04653r |  | increase | 2.13 | MYC-19-0166 | tL(UAG)1; tRNA-Leu, predicted by tRNAscan-SE; UAG anticodon |
| C | CAGL0C04697g |  | increase | 2.13 | MYC-19-0166 | Ortholog(s) have structural constituent of ribosome activity and mitochondrial small ribosomal subunit localization |
| D | CAGL0D01320g |  | increase | 2.13 | MYC-19-0166 | Ortholog(s) have non-membrane spanning protein tyrosine phosphatase activity, protein serine/threonine phosphatase activity, protein tyrosine phosphatase activity and role in protein dephosphorylation |
| E | CAGL0E01111g |  | increase | 2.18 | MYC-19-0166 | Ortholog(s) have role in actin filament-based process, mRNA 3'-end processing, mRNA export from nucleus, mitotic cell cycle and nuclear mRNA surveillance |
| E | CAGL0E01133g | *HOM2* | increase | 2.18 | MYC-19-0166 | Ortholog(s) have aspartate-semialdehyde dehydrogenase activity and role in homoserine biosynthetic process, methionine biosynthetic process, threonine biosynthetic process |
| E | CAGL0E01463g | *CDC33* | increase | 2.2 | MYC-19-0166 | Translation initiation factor eIF4E; protein abundance decreased in ace2 mutant cells |
| H | CAGL0H00330g |  | increase | 2.28 | MYC-19-0166 | Ortholog(s) have mitochondria-associated endoplasmic reticulum membrane localization |
| H | CAGL0H00352g |  | increase | 2.08 | MYC-19-0166 | Ortholog(s) have telomeric DNA binding activity, role in telomere capping, telomere maintenance via telomerase and chromosome, telomeric region, shelterin complex localization |
| H | CAGL0H02695g |  | increase | 2.06 | MYC-19-0166 | Ortholog(s) have glycogenin glucosyltransferase activity and role in glycogen biosynthetic process |
| I | CAGL0I10076r |  | increase | 2.34 | MYC-19-0166 | tA(AGC)7; tRNA-Ala, predicted by tRNAscan-SE; AGC anticodon |
| J | CAGL0J01353g |  | increase | 2.23 | MYC-19-0166 | Ortholog(s) have ATP binding, ATPase, metallopeptidase activity, role in protein-containing complex assembly, signal peptide processing and m-AAA complex, mitochondrial inner boundary membrane, mitochondrial inner membrane localization |
| J | CAGL0J08679g |  | increase | 2.19 | MYC-19-0166 | Ortholog(s) have role in cytoplasmic translation and cytoplasm localization |
| J | CAGL0J08712g |  | increase | 2.13 | MYC-19-0166 | Ortholog(s) have nicotinamide riboside transmembrane transporter activity, nucleobase transmembrane transporter activity, nucleoside transmembrane transporter activity |
| K | CAGL0K08712g |  | increase | 2.29 | MYC-19-0166 | Ortholog(s) have ribonuclease P activity, role in intronic box C/D RNA processing, tRNA processing and nucleolar ribonuclease P complex localization |
| L | CAGL0L10230g |  | increase | 2.04 | MYC-19-0166 | Ortholog(s) have protein-folding chaperone binding, protein-macromolecule adaptor activity |
| L | CAGL0L10252g |  | increase | 2.04 | MYC-19-0166 | Ortholog(s) have microtubule binding activity |
| M | CAGL0M07469g | *PEX12* | increase | 2.22 | MYC-19-0166 | Ortholog(s) have ubiquitin ligase activator activity, ubiquitin protein ligase activity and role in proteasome-mediated ubiquitin-dependent protein catabolic process, protein import into peroxisome matrix, protein polyubiquitination |
| M | CAGL0M07491g |  | increase | 2.22 | MYC-19-0166 | Ortholog(s) have role in cellular response to pheromone, protein deneddylation and COP9 signalosome localization |
| M | CAGL0M07634g | *EFG1* | increase | 2.12 | MYC-19-0166 | Transcription factor involved in control of biofilm formation |
| M | CAGL0M08822g | *HSP78* | increase | 2.37 | MYC-19-0166 | Ortholog(s) have ATPase, misfolded protein binding activity, role in cellular response to heat, mitochondrial genome maintenance, protein refolding, protein stabilization, protein unfolding and mitochondrial matrix localization |
| C | CAGL0C00814g |  | decrease | 0 | MYC-19-0181 | Protein of unknown function |
| D | **CAGL0D06512g** |  | increase | 2.1-2.19 | MYC-19-0238, MYC-19-0327 | Putative membrane bound guanine nucleotide exchange factor; gene is upregulated in azole-resistant strain |
| F | CAGL0F03575g |  | increase | 2.37 | MYC-19-0238 | Ortholog(s) have role in protein transport to vacuole involved in ubiquitin-dependent protein catabolic process via the multivesicular body sorting pathway and fungal-type vacuole localization |
| I | CAGL0I05082g |  | increase | 2.1 | MYC-19-0238 | Has domain(s) with predicted RNA binding, nucleic acid binding activity |
| I | CAGL0I09790g |  | increase | 2.09 | MYC-19-0238 | Ortholog(s) have role in endonucleolytic cleavage in 5'-ETS of tricistronic rRNA transcript (SSU-rRNA, 5.8S rRNA and LSU-rRNA) |
| F | CAGL0F06963g |  | increase | 2.05 | MYC-19-0273 | Ortholog(s) have microtubule binding, microtubule plus-end binding activity |
| I | CAGL0I09174g |  | increase | 2.04 | MYC-19-0273 | Ortholog(s) have histone H3K4 methyltransferase activity, role in chromatin remodeling and Set1C/COMPASS complex localization |
| J | CAGL0J11044g |  | increase | 2.06 | MYC-19-0273 | Ortholog of *S. cerevisiae*: PMT7, *C. albicans* SC5314: CR_09770C_A/PMT5, *C. dubliniensis* CD36: Cd36_34920, *C. parapsilosis* CDC317: CPAR2_701210 and *C. auris* B8441: B9J08_003692 |
| J | CAGL0J11176g |  | increase | 2.22 | MYC-19-0273 | Putative adhesin-like protein |
| L | CAGL0L01177g | *FRDS1* | increase | 3.25 | MYC-19-0330 | Putative soluble fumarate reductase |
| K | CAGL0K10736g | *CYB2* | increase | 2.02 | MYC-19-0334 | Ortholog(s) have L-lactate dehydrogenase (cytochrome) activity, role in lactate metabolic process and mitochondrial intermembrane space, mitochondrion localization |
| A | **CAGL0A00451g** | *PDR1* | increase | 2.02 | MYC-19-0345 | Zinc finger transcription factor, activator of drug resistance genes via pleiotropic drug response elements (PDRE); regulates drug efflux pumps and controls multi-drug resistance; gene upregulated and/or mutated in azole-resistant strains |
| H | CAGL0H02607g | *SPE2* | increase | 2.15 | MYC-19-0345 | Ortholog(s) have role in spermidine biosynthetic process, spermine biosynthetic process |
| H | CAGL0H05599g |  | increase | 2.15 | MYC-19-0345 | Ortholog(s) have dihydroceramidase activity, role in ceramide biosynthetic process, ceramide catabolic process and endoplasmic reticulum localization |

Chr, Chromosome.

^a^Boldface indicates genes implicated in azole resistance in *C. glabrata*.

^b^Descriptions sourced from *Candida* Genome Database.
